# Structures of the neutral amino acid transporter LAT4 provide insights into antitumor effects of its inhibitor tubeimoside-1

**DOI:** 10.1038/s44318-026-00786-0

**Published:** 2026-05-04

**Authors:** Dian Ding, Yishuo Lu, Jingyi Yang, Hongyi Chen, Peijun Jiang, Yan Jin, Jianyuan Luo, Guangxi Wang, Yuxin Yin

**Affiliations:** 1 Department of Biomedical Sciences, Division of Biomedical Health Sciences, School of Medicine, The Chinese University of Hong Kong, Shenzhen, 518172 China; 2 Institute of Systems Biomedicine, Department of Pathology, Beijing Key Laboratory of Tumor Systems Biology, Peking-Tsinghua Center for Life Sciences, School of Basic Medical Sciences, Peking University Health Science Center, Beijing, 100191 China; 3 Department of Medical Genetics, School of Basic Medical Sciences, Peking University Health Science Center, Beijing, 100191 China; 4 Institute of Precision Medicine, Peking University Shenzhen Hospital, Shenzhen, 518036 China

**Keywords:** Membranes & Trafficking, Structural Biology

## Abstract

Methionine restriction has emerged as a promising strategy for extending lifespan and enhancing cancer therapy. LAT4, an amino acid transporter encoded by SLC43A2, is frequently overexpressed in multiple cancers and critically contributes to systemic methionine accumulation. However, the structural basis of LAT4 function remains poorly understood, and no effective inhibitors have been developed to date. In this study, we present high-resolution cryo-electron microscopy structures of LAT4 and the related SLC43A3-encoded purine transporter ENBT1. The phenylalanine-bound structure of LAT4 enables the characterization of the substrate binding pocket. Comparison of the outward-facing ENBT1 and inward-facing LAT4 structures identifies key residues involved in the methionine transport process. Structural analysis of digitonin binding to the central cavity of LAT4 enabled identification of tubeimoside-1 (TBM-1) as a potent inhibitor of LAT4-mediated methionine uptake. We demonstrate that tubeimoside-1 reduces methionine uptake in B16F10 cancer cells. Furthermore, TBM-1 suppresses tumor progression in the MMTV-PyVT mouse model of breast cancer through systemic methionine restriction. Our study provides insights into the LAT4 transport mechanism and identifies tubeimoside-1 as a potent inhibitor of methionine uptake and establishes a foundation for developing LAT4-targeting therapeutics to restrict methionine uptake.

## Synopsis

The amino acid transporter LAT4 is overexpressed in cancer and contributes to tumorigenesis by promoting methionine accumulation. This study solves cryo-EM structures of LAT4 and the related transporter ENBT1, providing insights into the molecular basis of methionine transport and enabling identification of tubeimoside-1 as a LAT4 inhibitor.

- High-resolution cryo-EM structures of LAT4 and ENBT1 reveal key conformational states and residues governing methionine transport.
- The phenylalanine-bound LAT4 structure reveals the substrate-binding pocket.
- Molecular dynamics simulations and functional analyses identify key amino acid residues critical for substrate recognition.
- Digitonin binding to LAT4 hydrophobic cavity enables the identification of tubeimoside-1 as a potent inhibitor of LAT4-mediated methionine uptake.

## Abstract (web summary)

Cryo-electron microscopy structures of LAT4 and the related purine transporter ENBT1 reveal the key features of substrate transport via LAT4 and its inhibition by saponins.

## Graphical abstract

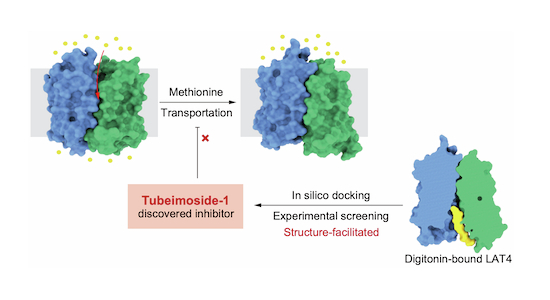

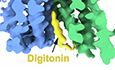

## Introduction

Methionine is an essential amino acid in humans (Mastrototaro et al, 2016). Its metabolism is involved in many cellular functions, including protein translation, cysteine biosynthesis, as well as reduced glutathione (GSH) production. In addition, methionine is the main cellular donor of methyl groups after its conversion to S-adenosylmethionine (SAM), which is key to controlling the entire cellular transcriptome by serving as a methyl donor for DNA methylation (Chiang et al, 1996; Mastrototaro et al, 2016). Although methionine is essential for whole-body growth and development, continuous high intake of this amino acid may be detrimental (Mastrototaro et al, 2016). Hypermethioninemia has been associated with liver damage (Labrune et al, 1990), neurological disorders (Chamberlin et al, 1996), increased vascular risk and exacerbation of psychopathological symptoms in schizophrenic patients (Garlick, 2006). Dietary methionine restriction (MR) provides metabolic benefits such as extending lifespan (Lee et al, 2014), reducing adiposity (Wanders et al, 2018) and increasing insulin sensitivity (Stone et al, 2014, Wanders et al, 2017; Wanders et al, 2020). The requirement of cancer cells for methionine differs from that of normal cells (Yamamoto et al, 2022). Thus, MR has been shown to induce apoptosis in human prostate cancer cell lines (Fu et al, 2003; Fu et al, 2006), decrease cell proliferation and increase apoptosis in breast cancer tissues (Hens et al, 2016), and enhance the efficacy of chemotherapeutic agents in patient-derived xenograft models of colorectal cancers (Gao et al, 2019). This so-called methionine addiction of cancer cells makes MR an attractive approach as a potential cancer treatment (Wanders et al, 2020).

Methionine must be obtained from the diet, and its absorption from the gastrointestinal tract is highly efficient (Webb, 1990). Methionine absorption across the intestinal epithelium is mainly mediated by Ace2/B^0^AT1 on the apical membrane and LAT4 (SLC43A2) on the basolateral membrane (Broer and Fairweather, 2018). LAT4 is also important for methionine reabsorption in the kidney, and knockout of the *Lat4* gene leads to an early lethal malnutrition-like phenotype in the mouse (Guetg et al, 2015; Rajendran et al, 2020). LAT4 belongs to the L‑type amino acid transporter family, which comprises LAT1 and LAT2 from the SLC7 subfamily, and LAT3 and LAT4 from the SLC43 subfamily (Wang & Holst, 2015). The SLC43 family consists of three members: LAT3 (SLC43A1), LAT4 (SLC43A2), and ENBT1 (SLC43A3) (Bodoy et al, 2013). Human SLC43A2 (LAT4) shares 59% amino acid identity with human SLC43A1 (LAT3) and 31% identity with human SLC43A3 (ENBT1) (Appendix Fig. S1A). LAT4 transports a narrow range of neutral amino acids, including methionine, phenylalanine, leucine, isoleucine and valine, through facilitated diffusion (Bodoy et al, 2005). The role of LAT4 as a methionine transporter in several types of cancer has been reported in recent studies (Bian et al, 2020; Gubser and Kallies, 2020; Pandit et al, 2023). Cancer cells with increased LAT4 expression consumed and outcompeted T cells for methionine, which in turn impaired T cell immunity by disrupting methionine metabolism, increasing PD-1 expression in tumor-infiltrating CD4^+^ T cells and decreasing intracellular SAM in CD8^+^ T cells (Bian et al, 2020; Pandit et al, 2023). Downregulation of LAT4 using a CRISPR/Cas9 gene knockout system relieved the competitive pressure of methionine on T cells and promoted CD8^+^ T cell infiltration in tumors (Huang et al, 2023). MR is a promising therapeutic strategy for cancer treatment due to its inhibitory effect on tumor proliferation and its capacity to enhance antitumor immunity. In parallel, the influence of LAT4 expression within the tumor microenvironment on metabolic reprogramming remains to be elucidated. However, progress in this area is hindered by the lack of detailed structural insights into the LAT4 transport mechanism and the absence of potent LAT4 inhibitors. Developing an accurate structural model of LAT4 will be essential to facilitate the rational design of pharmaceuticals targeting this transporter.

Here, we report a series of SLC43 family transporter structures determined by cryo-electron microscopy (cryo-EM), which reveal the substrate-binding pockets of LAT4. We also show how compounds derived from natural sources can inhibit LAT4 activity and cancer progression. Together with functional analyses and molecular dynamics simulations, these structures provide mechanistic insights into the LAT4 substrate transport mechanism as well as inhibition. Our findings should advance the future development of potential MR drugs targeting LAT4.

## Results

### Structural determination and overall architecture of LAT4

**Figure 1:**
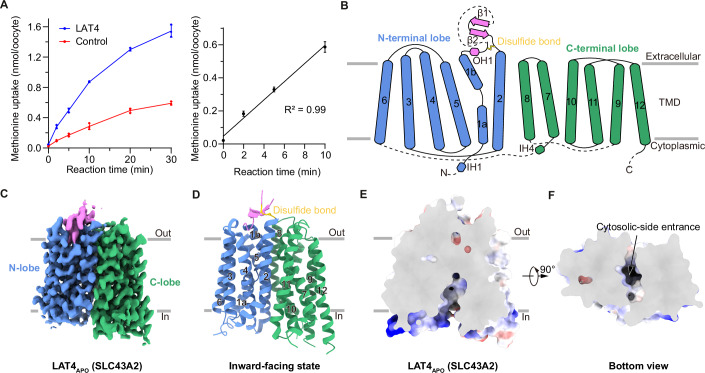
Activity and structures of human LAT4. (**A**) Time course of [^3^H]methionine (0.5 mM, 10 μCi) accumulation in oocytes. Accumulation in oocytes injected with and without (control) cRNA encoding wild-type human LAT4 (left panel). Methionine uptake by hLAT4 (accumulation of injected oocytes minus non-injected oocytes) over 10 min is linear (right panel). Data points are means ± SD (*n* = 3 biologically independent experiments). (**B**) Topology of the secondary structure of hLAT4. N-terminal lobe (N-lobe), C-terminal lobe (C-lobe) and extracellular loops shown in blue, green and pink, respectively. Unresolved regions are shown as dashed lines. The disulfide bond is shown as yellow sticks. The number of transmembrane helices (TM) are labeled as above. TMD, transmembrane domain. (**C**) Cryo-EM density of hLAT4_APO_ with its N-terminal lobe (N-lobe), C-terminal lobe (C-lobe) and extracellular loops colored as in (**B**). (**D**) The overall structure of hLAT4_APO_ in the inward-facing conformation is colored as in (**B**). The extracellular disulfide bond is shown as yellow sticks. The number of the TMs are labeled as above. (**E**,** F**) Cut-open side view (E) and cytosolic view (**F**) of the central cavity of hLAT4. The surface of hLAT4 is colored according to the electrostatic potential calculated using ChimeraX. Source data are available online for this figure.

Xenopus laevis oocytes injected with human LAT4 cRNA exhibit time-dependent accumulation of [^3^H]methionine (Fig. 1A). Full-length wild-type hLAT4 fused with a C-terminal affinity tag was then expressed in HEK293F cells (Goehring et al, 2014). Recombinant LAT4 protein was extracted from membranes using lauryl maltose neopentyl glycol (LMNG) and cholesteryl hemisuccinate (CHS). LAT4 proteins were reconstituted into nanodiscs and purified using size exclusion chromatography (SEC) to avoid the problem of preferred particle orientation (Appendix Fig. S1B,C). Protein from the peak fractions was absorbed onto fabricated grids coated with a reduced graphene oxide membrane (Liu et al, 2021). The apo structure of LAT4 (termed LAT4_APO_) was determined after cryo-EM data collection and image analyses with reconstruction at an overall resolution of 2.9 Å estimated by the gold-standard Fourier shell correlation (Appendix Fig. S2C–F). All 12 transmembrane (TM) helices are well resolved in the LAT4_APO_ structure (Appendix Fig. S2G). The transmembrane domain (TMD) of human LAT4 exhibits the topology of the canonical major facilitator superfamily (MFS) fold (Drew et al, 2021). The 12 TM segments form two bundles, the N-terminal lobe (TM1–TM6) and the C-terminal lobe (TM7–TM12) (Fig. 1B–D). Surface analysis showed that LAT4_APO_ is arranged in an inward-facing conformation with a central cavity accessible from the cytoplasmic side (Fig. 1E,F). The electrostatically neutral, solvent-accessible cavity is mainly surrounded by TM2, TM4, TM5, TM8, TM10, and TM11 (Fig. 1E,F). The extracellular domain (ECD) of LAT4 consists of a 38 amino acid linker that connects TM1 to TM2. Its structure contains a short helix and an anti-parallel β-sheet; however, amino acids 53–71 of the ECD were not resolved, probably due to their flexibility (Appendix Fig. S2G). The ECD in the inward-facing state of LAT4 interacts with the N-terminal lobe through hydrophobic interactions (between F45, L40 and I147) and hydrogen bonds (between D85, S80, Q84, F45, Y46, and S144). The structure of the ECD is also stabilized by the disulfide bond formed between C50 and C81 (Appendix Fig. S2M). The intracellular loop of LAT4 contains a large linker sequence connecting TM6 to TM7 (residues 231–313), which was not resolved in the LAT4_APO_ state, probably because of its dynamic nature.

### Substrate translocation pathway of LAT4

**Figure 2:**
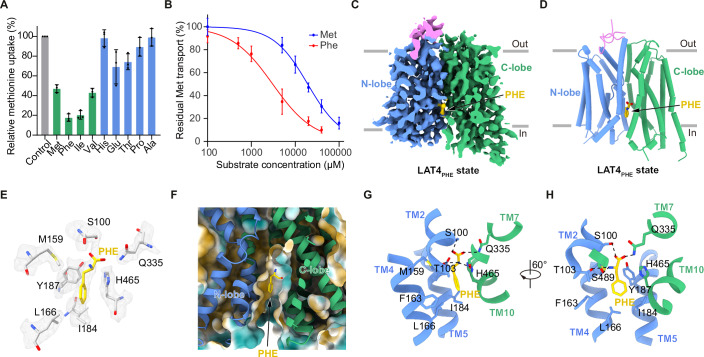
Phenylalanine binding pocket of LAT4. (**A**) Competition assay for [^3^H]methionine (0.5 mM, 10 μCi) transport by wild-type LAT4 at 10 min in the presence of the indicated amino acids (20 mM). Competing and non-competing amino acids are colored green and blue, respectively. Data shown are means ± SD (*n* = 3 biologically independent experiments). (**B**) Effect of phenylalanine and methionine on [^3^H]methionine (10 μCi) uptake by LAT4 in oocytes. The IC_50_ values for phenylalanine and methionine were determined to be 3.06 mM and 19.21 mM, respectively. Data shown are mean values ± SD (*n* = 3 biologically independent experiments). (**C**,** D**) Side view of the cut-open cryo-EM density (**C**) and structure (**D**) of LAT4 in complex with phenylalanine. The N-lobe, C-lobe, extracellular loops, and phenylalanine are shown in blue, green, pink, and yellow, respectively. Phenylalanine is shown as sticks. (**E**) Cryo-EM density of phenylalanine at the substrate-binding site was further sharpened with a B factor of −60 Å^2^ in Coot, contoured at 6.1 σ and shown as gray meshes. Phenylalanine is shown as yellow sticks, and the side chains of the surrounding residues are shown as gray sticks. (**F**) The hydrophobicity surface of the permeation pathway of LAT4 in the inward-facing state. Phenylalanine is shown as yellow sticks. The hydrophobic surface (formed by TM1, TM4, and TM5) and the hydrophilic surface (formed by TM2, TM7, and TM11) of LAT4 are colored in blue and yellow, respectively, as calculated using ChimeraX. (**G**,** H**) Close-up views of the phenylalanine-binding site boxed in (**F**). Residues interacting with phenylalanine are shown as sticks, and hydrogen bonds are indicated by dashed lines. Phenylalanine is shown as yellow sticks. Source data are available online for this figure.

Cold-competition experiments showed that [^3^H]methionine uptake was inhibited by extracellular amino acid substrates at 20 mM; methionine and phenylalanine inhibited methionine transport by LAT4 with IC_50_ values of 19.21 and 3.06 mM, respectively (Fig. 2A,B). To investigate the atomic determinants of LAT4 substrate recognition and transport mechanism, the structure of LAT4 in complex with phenylalanine instead of methionine was determined because of the relatively higher selectivity of LAT4 for the former amino acid (Fig. 2B). A LAT4 structure in complex with phenylalanine (LAT4_PHE_) was obtained at an overall resolution of 3.2 Å estimated by gold-standard Fourier shell correlation (Appendix Fig. S3A–F). In this structure, LAT4_PHE_ adopted an inward-facing conformation which closely resembles the LAT4_APO_ structure with a root mean squared deviation (r.m.s.d) value of 0.948 Å. Non-protein densities, which were absent in LAT4_APO_, were observed within the central cavity in the cryo-EM map of LAT4_PHE_ (Fig. 2C,E). Phenylalanine binds at the end of the substrate translocation pathway (Fig. 2C,D) which consists of bifacial surfaces: one hydrophobic surface lined by TM1b, TM4 and TM5, and one hydrophilic surface lined by TM2, TM10, and TM11 (Fig. 2F). The side chains of Y187 and H465 narrow the central binding pocket of phenylalanine (Fig. 2G,H). The phenyl side chain of phenylalanine engages in a π–π interaction with the aromatic ring of Y187 and forms hydrophobic contacts with the imidazole group of H465, whereas the carboxyl and amino groups of phenylalanine form hydrogen bonds with S100 and Q335. A hydrophobic pocket formed by M159, F163, L166, and I184 may also contribute to the substrate preference of LAT4 for phenylalanine (Fig. 2G,H).

### The LAT4 methionine binding site

**Figure 3:**
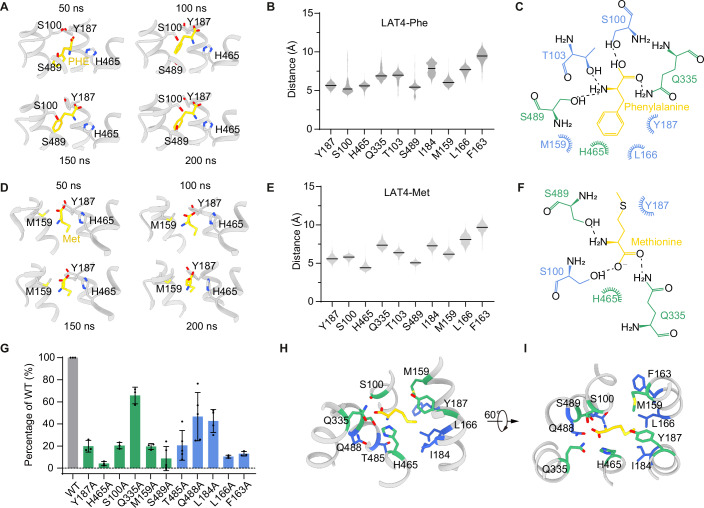
MD simulations of the methionine binding pocket of LAT4. (**A**) Representative snapshots for MD simulations of phenylalanine binding to hLAT4 at regular intervals of 50 ns. Phenylalanine interacting residues are colored in gray and shown as sticks. Phenylalanine is shown as yellow sticks. (**B**) The distances between the center of mass of phenylalanine and its interacting residues forming the central binding pocket in 200 ns MD simulations. (**C**) Ligplot schematic of the interactions between phenylalanine and LAT4. Phenylalanine, its interacting residues in the N-lobe and the C-lobe are colored in yellow, blue and green, respectively. (**D**) Representative snapshots for MD simulations of methionine binding to hLAT4 at regular intervals of 50 ns. Methionine interacting residues are colored in grey and shown as sticks. Methionine is shown as yellow sticks. (**E**) The distances between the center of mass of methionine and its interacting residues forming the central binding pocket in 200 ns MD simulations. (**F**) Ligplot schematic of the interactions between methionine and LAT4. methionine, its interacting residues in the N-lobe and the C-lobe are colored in yellow, blue and green, respectively. (**G**) Effect of alanine substitutions of key residues in the central binding site on LAT4 [^3^H]methionine (0.5 mM, 10 μCi) uptake activity. Residues that consist of the methionine binding pocket and the translocation pathway are colored in green and blue, respectively. Data were expressed relative to wild-type LAT4, and the bars represent mean values ± SD (*n* ≥ 3 biologically independent experiments). (**H**,** I**) Predicted interactions between methionine and residues of LAT4 at the 200 ns time point of the MD simulations. Source data are available online for this figure.

The substrate-binding residues within the LAT4 cavity are highly conserved among LAT4 orthologues (Appendix Fig. S1A). To gain insights into the methionine recognition mechanism, we performed molecular dynamics (MD) simulations of LAT4_PHE_ as a control (Fig. 3A; Appendix Fig. S3H,I). We analyzed the distance distributions from the center of mass for phenylalanine and surrounding LAT4 residues and found that S100, Y187, H465, and S489 make the closest contacts (Fig. 3B,C). The phenylalanine stays in the pocket through stable interactions with surrounding residues further confirms the key role of these residues in the coordination of substrates (Figs. 2G,H and 3A). We next replaced the phenylalanine in the LAT4 central cavity with methionine and performed MD simulations over 200 ns. Methionine remained statically bound in the phenylalanine-binding pocket, indicating that these substrates of LAT4 share a similar binding pocket (Fig. 3D,H,I; Appendix Fig. S3J). Methionine may form hydrogen bonds with S100, Q335, and S489 (Fig. 3E,F).

To assess the functional role of residues in the substrate translocation pathway and binding pocket of LAT4, a series of mutants were generated, and their functionality were assessed by measuring the uptake of radiolabeled methionine after expression in *Xenopus laevis* oocytes. Alanine substitutions of the residues closest to methionine (S100, Y187, H465, and S489) substantially reduced methionine transport by LAT4 (by >80%) without significantly affecting its expression or membrane trafficking (Fig. 3G; Appendix Fig. S2N). In addition, alanine substitution of amino acids along the substrate translocation pathway (T485, Q488, F163, L166, and I184) caused a 30–90% reduction in methionine transport (Fig. 3G). These results suggest that disruption of the hydrogen bonds and hydrophobic interactions between methionine and residues along the substrate permeation pathway may also reduce LAT4 activity.

### Mechanism of LAT4 conformation rearrangement

**Figure 4:**
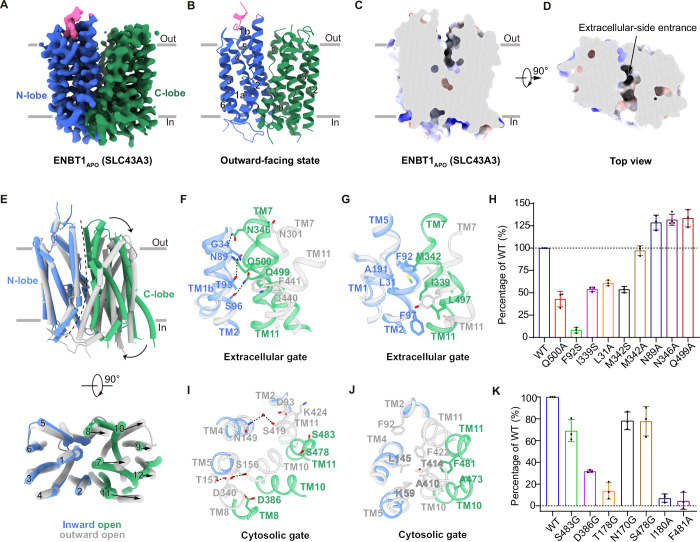
Conformational changes between different states of the SLC43 transporters hENBT1 and hLAT4. (**A**) Cryo-EM density of hENBT1_APO_ with its N-lobe, C-lobe and extracellular loops colored blue, green and pink, respectively. (**B**) The overall structure of hENBT1_APO_ in the outward-facing conformation is colored as in (**A**). The TM helices are numbered as above. (**C**,** D**) Cut-open side view (**C**) and extracellular view (**D**) of the central cavity of hENBT1. The surface of hENBT1 is colored according to electrostatic potential as calculated using ChimeraX. (**E**) Comparison of the inward-facing hLAT4_APO_ (colored green and blue) with the outward-facing hENBT1_APO_ (white) as aligned by their N-lobes and viewed from the side (upper) and the extracellular face (lower). The arrows indicate the movement of the C-lobe from an inward-facing to an outward-facing conformation. The TM helices are numbered as above. (**F**,** G**) Hydrophilic (**F**) and hydrophobic (**G**) interactions between the N-lobe and the C-lobe of inward-facing hLAT4_APO_. Putative polar interactions are depicted as dashed lines. LAT4 and ENBT1 are colored as in (**E**). (**H**) [^3^H]methionine (0.5 mM, 10 μCi) transport activity of wild-type LAT4 and its inward-facing destabilizing mutants. Data were expressed relative to wild-type LAT4 activity, and the bars represent mean values ± SD (*n* ≥ 3 biological independent experiments). (**I**,** J**) Hydrophilic (**I**) and hydrophobic (**J**) interactions between the N-lobe and the C-lobe of outward-facing hENBT1_APO_. Putative polar interactions are depicted as dashed lines. LAT4 and ENBT1 are colored as in (**E**). (**K**) [^3^H]methionine (0.5 mM, 10 μCi) transport activity of wild-type LAT4 and its outward-facing destabilizing mutants. Data were expressed relative to wild-type LAT4 activity, and the bars represent mean values ± SD (*n* ≥ 3 biological independent experiments). Source data are available online for this figure.

To study the structural changes of LAT4 that occur during its substrate transport process, recombinant ENBT1 protein was also extracted from membranes using LMNG and CHS and purified using SEC (Appendix Fig. S1D,E). The apo structure of ENBT1 (termed ENBT1_APO_) was then determined at an overall resolution of 3.2 Å, with 12 TM helices well resolved (Appendix Fig. S2H–L). ENBT1_APO_ is arranged in an outward-facing conformation with its central cavity accessible from the extracellular face of the membrane (Fig. 4A–C), and the solvent-accessible cavity is mainly surrounded by TM1b, TM2, TM5, TM7, TM8, and TM11 (Fig. 4D).

MFS-fold transporters typically use a complex rocker-switch model of transport involving both domain rotation and local structural rearrangements (Yan, 2015). Two helical bundles transit from an outward-facing to an inward-facing conformation to form cavity-closing contacts during the translocation of substrate (Drew et al, 2021). Comparison of the inward-facing LAT_APO_ with the outward-facing ENBT1_APO_ by aligning their N-terminal lobes illustrates the movements of the C-terminal lobe (Fig. 4E). The N-terminal and C-terminal lobes of LAT4_APO_ exhibit a high degree of structural similarity to ENBT1_APO_, with RMSD values of 1.268 Å for the N-terminal lobe and 1.363 Å for the C-terminal lobe.

TM1b, TM2, and TM5 of the N-terminal lobe and TM7, TM8, and TM11 of the C-terminal lobe of LAT4 form the interface between the two lobes in the inward-facing state (Fig. 4E). Hydrogen bonds form between Q500 and the side chains of T93 and N89, and between Q499 and S96 at the TM2–TM11 interface. At the TM1b–TM7 interface, the side chain of N346 forms hydrogen bonds with the backbone of G34 (Fig. 4F). Hydrophobic residues of TM1 (L31), TM2 (F92, F97), TM5 (A191), TM7 (I339 and M342), and TM11 (L497) of LAT4 stabilize the extracellular gate and prevent the extracellular translocation of amino acid substrates (Fig. 4G). Residues involved in these conformational changes are well conserved in SLC43 family transporters Appendix Fig. S1A). Mutagenesis studies of residues forming the extracellular gate showed that single alanine substitutions of Q500 and L31, as well as single serine substitutions of F92, I339, and M342, substantially reduced LAT4 methionine transport activity (Fig. 4H). Single alanine substitutions of N89, N346 and Q499 enhanced the transport activity of LAT4, whereas alanine substitution of M342 did not. However, serine substitution of M342 caused a 50% decrease in activity, suggesting that hydrophobic interactions are essential for LAT4 to form the inward-facing conformation and their disruption may disrupt the transport cycle (Fig. 4H).

TM2, TM4, and TM5 of the N-terminal lobe and TM8, TM10 and TM11 form the cytosolic gate in the outward-facing state of ENBT1 (Fig. 4E). Hydrogen bonds form among T157, D340, and S156 and among N149, S419, of ENBT1 and a water molecule (Fig. 4I). F92, L145, I159, A410, T414, and F422 of ENBT1 form the hydrophobic interface (Fig. 4J). Mutagenesis analysis of corresponding residues in LAT4 suggests hydrogen bonds (involving T178-D386-S177, N170-S478, and D111-S483) and hydrophobic interactions (M110, L166, I180, A473, and F481) formed between TM2–TM11 and TM5–TM8–TM10 stabilize the LAT4 cytosolic gate (Fig. 4K). Distinct from pH-sensitive MFS members (Schmiege et al, 2024; Shan et al, 2023; Wu et al, 2024), no salt bridge is observed at the interface between the N- and C-terminal lobes of LAT4, which is consistent with its pH-insensitive property (Bodoy et al, 2005).

### Identification of LAT4 inhibitors

**Figure 5:**
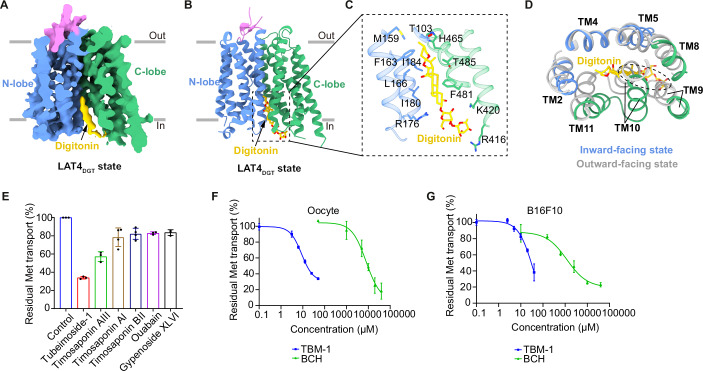
Tubeimoside-1 is an inhibitor of LAT4-dependent methionine transport. (**A**) A cut-open side view of the cryo-EM density of LAT4 in the digitonin-bound state contoured at 6.9 σ. Digitonin is colored in yellow, and LAT4 is colored with its N-lobe, C-lobe and extracellular loops in blue, green and pink, respectively. (**B**) Cartoon representation of LAT4 in the digitonin-bound state colored as in (**A**). Digitonin (boxed) is shown as yellow sticks. (**C**) Close-up view of the digitonin-binding site boxed in (**B**). Digitonin (boxed) is shown as yellow sticks. Residues surrounding the digitonin molecule are shown as sticks and colored as in (**A**). (**D**) Comparison of inward-facing hLAT4_DGT_ (colored green and blue) with outward-facing hENBT1_APO_ (white) aligned according to their N-lobes. Steric clashes between digitonin and the TMs of the outward-facing state of hENBT1_APO_ are outlined by dashed circles. Digitonin is shown as yellow sticks. (**E**) Inhibitory effects of saponins (at 50 μM) on the [^3^H]methionine (0.5 mM, 10 μCi) uptake activity of LAT4 in an oocyte expression system. Data shown are expressed relative to wild-type hLAT4 activity in the absence of inhibitor. The bars represent mean values ± SD (*n* ≥ 3 biological independent experiments). (**F**) Concentration response curves showing the inhibitory effects of BCH and tubeimoside-1 (TBM-1) on the [^3^H]methionine (0.5 mM, 10 μCi) uptake activity of LAT4 in oocytes. The IC_50_ values of BCH and TBM-1 were calculated to be 8.02 mM and 8.83 μM, respectively. Data points are mean values ± SD (*n* ≥ 3 biological independent experiments). (**G**) Concentration-dependent inhibition of [^3^H]methionine (0.5 mM, 10 μCi) uptake in B16F10 cells by BCH and TBM-1. Data were expressed relative to uptake in cells without the addition of inhibitors. Data points are mean values ± SD (*n* ≥ 3 biological independent experiments). Source data are available online for this figure.

While optimizing the cryo-EM sample preparation process for LAT4 to improve the protein signal, 0.1% (w/v) digitonin was used, and the structure was determined at an overall resolution of 3.8 Å (Appendix Fig. S4). In the cryo-EM map of LAT4_DGT_, an elongated density for a digitonin molecule was observed parallel to TM4 and TM5 (Fig. 5A; Appendix Figs. S4F,G and S5A). The sugar moieties of digitonin were less clearly resolved, probably due to their flexibility (Appendix Fig. S4G). The digitogenin moiety of digitonin extended into the inner part of the central cavity and formed extensive contacts mainly with the hydrophobic side of the substrate permeation pathway, which is lined by the side chains of F163, L166, I180, I184, and F481 (Fig. 5B,C). The binding site of digitonin partially overlaps with the amino acid recognition pocket (Appendix Fig. S5B) and restricts the rearrangements of the TM helices and the formation of the cytosolic gate in the outward-facing conformation (Fig. 5D). Molecular docking (Friesner et al, 2004) and MD simulations of a complete digitonin molecule using the LAT4_DGT_ structure, with the digitogenin group stably bound in the central cavity, further support our model (Appendix Fig. S5C–E). Therefore, we hypothesize that interactions between digitonin and the transmembrane domain could block the substrate permeation pathway and the conformational changes of LAT4, and consequently, attenuate its substrate transport activity.

Digitonin is a natural saponin extracted from the foxglove plant, *Digitalis purpurea*. To explore the effectiveness of structurally similar saponins in inhibiting LAT4 and their potential for MR, molecular docking was performed between mild saponins (less disruptive to the plasma membrane) and LAT4 (Appendix Fig. S5F). The inhibitory effects of these compounds on LAT4 transport activity were screened using a methionine uptake assay, and the results show that LAT4-mediated uptake was inhibited 20–70% by these saponins when tested at 50 μM (Fig. 5E). Among the molecules tested, Tubeimoside-1 (TBM-1) exhibited the greatest inhibition of LAT4 transport activity. Subsequent concentration response experiments showed that the IC_50_ of TBM-1 is 1000-fold lower than the IC_50_ of BCH (the only known LAT4 inhibitor) in LAT4-expressing oocytes (Fig. 5F). TBM-1 inhibited methionine uptake in B16F10 cancer cells and exhibited substantially greater potency than BCH (Fig. 5G). TBM‑1 inhibits LAT4 in a non‑competitive manner, markedly reducing Vmax from 2.23 to 0.63 nmol/min upon TBM‑1 treatment, with minimal effects on *K*_m_ (8.39 vs 8.21 mM in control and TBM‑1‑treated groups, respectively) (Appendix Fig. S5G). As TBM-1 is considerably more potent than the previously known LAT4 inhibitor BCH, it will serve as a superior lead compound for designing better LAT4 inhibitors that will facilitate studies of the effect of MR and its potential as an anti-cancer therapy.

### LAT4 inhibition by TBM-1 restrains breast cancer progression in MMTV-PyVT mice

**Figure 6:**
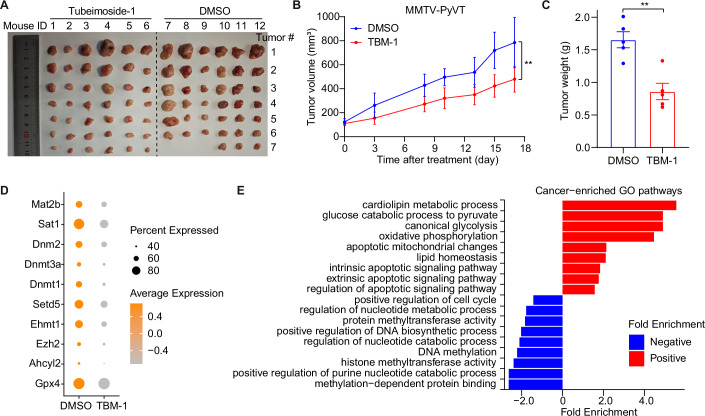
LAT4 inhibition by TBM-1 restrains breast cancer progression in MMTV-PyVT mice. (**A**) Representative images of mammary tumors isolated from MMTV-PyVT female mice following intraperitoneal injection of DMSO (control) or TBM-1 (4 mg/kg daily) for 3 weeks. (**B**) Tumor growth curves showing cancer progression in MMTV-PyVT mice over 3 weeks of DMSO or TBM-1 treatment. Data shown are means ± SEM (*n* = 5 biological replicates; *p* = 0.0038 by paired *t*-test). (**C**) Quantification of the final mammary tumor weights of the five largest tumors from MMTV-PyVT mice treated for 3 weeks with DMSO (control) and TBM-1 at the end of the treatment. Data shown are means ± SEM (*n* = 5 biological replicates; *p* = 0.002 by two-tailed *t*-test). (**D**) Dot plot showing the expression levels of representative genes associated with methionine metabolism in breast cancer cells from the DMSO (control) and TBM-1-treated mice. (**E**) GO enrichment analysis of differentially expressed genes (DEGs) in breast cancer cells from TBM-1-treated mice compared to DMSO-treated controls. Nine representative positively enriched GO pathways (fold enrichment >0, *q* value <0.05) and nine representative negatively enriched GO pathways (fold enrichment <0, *q* value <0.05) are shown. Source data are available online for this figure.

To further explore the systemic MR effects of TBM-1 on tumor progression, we utilized the MMTV-PyVT mouse model, which spontaneously develops synchronous multifocal tumors in all mammary glands (Liu et al, 2023). Intraperitoneal injection of TBM-1 at a daily dose of 4 mg/kg for 3 weeks significantly reduced tumor volume and tumor weight of about one-half compared to mice receiving only DMSO (vehicle control) (Fig. 6A–C).

To explore the potential tumor suppression mechanisms of TBM-1, we performed single-cell RNA sequencing (scRNA-seq) on cells isolated from the breast cancer tissues of DMSO- and TBM-1-treated mice. Gene expression profiles of 15,800 cells were retained after quality control filtering. The cells were grouped into ten major clusters, each annotated according to canonical markers as described by Kumar et al, (2023) (Appendix Fig. S6A,B). Breast cancer cells were further validated by calculating potential copy number variations (CNVs) using the “inferCNV” package (Appendix Fig. S6C). The cell compositions of the DMSO control and TBM-1 groups were generally comparable for most cell types, such as LumSec, LumHR, basal and adipocytes. However, immune cells, especially myeloid and T cells, were significantly increased in the TBM-1 group (Appendix Fig. S6D). TBM-1 administration significantly inhibited methionine-involved pathways, evidenced by the downregulation of genes associated with methionine metabolism (Mat2b), polyamine metabolism (Sat1), DNA methylation (Dnm2, Dnmt3a, and Dnmt1), histone methylation (Setd5, Ehmt1, and Ezh2), and oxidative stress resistance (Ahcyl2 and Gpx4) (Fig. 6D), leading to negative enrichment in methylation pathways and DNA biosynthetic process in cancer cells from the TBM-1 treated group, reviewed by results from the gene ontology (GO) analysis (Fig. 6E). GO analysis also showed that breast cancer cells from TBM-1-treated mice were positively enriched in apoptotic signaling pathways, while negatively enriched in various cancer progression pathways, including cell cycle (Fig. 6E). Collectively, these findings suggest that TBM-1 induces MR and suppresses cancer progression in the MMTV-PyVT breast cancer model.

## Discussion

Methionine is an essential amino acid important for cancer cell growth (Wanders et al, 2020). The reliance of cancer cells on exogenous methionine renders MR a promising strategy in cancer therapy (Ji et al, 2024). Preclinical trials indicate that MR by diet or methioninase produces antitumor effects, especially when combined with chemotherapy (Chaturvedi et al, 2018; Yamamoto et al, 2022). In this study, we determined the Phe-bound structure of LAT4, one of the critical membrane transporters for systemic methionine accumulation and conducted MD simulations alongside functional analyses to validate our model of the substrate-binding pocket. Phe and Met share a similar binding site, which consists of Y187, H465, S100, Q335, and S489 (Fig. 3A,C). Hydrophobic residues along the permeation pathway also contribute to the substrate transport process (Figs. 2G and 3F). Structural comparison of the inward-facing LAT4 with the outward-facing ENBT1, along with functional analysis, identified the key residues involved in the conformational changes of LAT4 that occur during substrate binding and transport (Fig. 4).

The structure of digitonin-bound LAT4 provides insights into the potential inhibitory effects of saponins on LAT4-dependent methionine transport and may help facilitate the future design of agents for MR therapy. Distinct from its binding site at the surface of other transporters (Rodriguez et al, 2021; Rullo-Tubau et al, 2024), digitonin localizes to the substrate permeation pathway of inward-facing LAT4 mainly through interactions between the digitogenin moiety and a hydrophobic patch of the N-terminal lobe of LAT4 (Fig. 5B,C). This interaction prevents methionine from entering the central cavity and precludes the structural transition of LAT4 and hence transport activity (Fig. 5D; Appendix Fig. S5B). These results provided a molecular basis for the screening of structurally similar saponins which led to the identification of TBM-1 as an effective inhibitor of LAT4 (Fig. 5E). Natural compounds have long been known to possess therapeutic efficacy in a broad variety of solid cancers, including breast, lung, colorectal and cervical carcinomas (Jiang et al, 2019; Shen et al, 2024; Sy et al, 2008; Wang et al, 2020). Among these natural compounds, TBM-1 has demonstrated significant antitumor effects through multiple different pathways (Feng et al, 2018; Peng et al, 2016; Song et al, 2023). Our findings identify LAT4 as a target for TBM-1 and provide a new approach for MR therapy. By administering TBM-1 to MMTV-PyVT breast cancer mice, we showed that this compound attenuates tumor progression, even in tumors that may not primarily rely on LAT4 for methionine uptake (Fig. 6A–C; Appendix Fig. S6B,D). TBM-1 treatment impairs methionine-dependent biochemical pathways essential for cancer progression (Fig. 6D,E), suggesting LAT4 not only as a potential target within tumors but also as a systemic MR target in non-tumor tissues. This positions TBM-1 as a prototype for the development of LAT4-directed MR therapeutics.

## Methods

**Table:** Reagents and tools table

Reagent/resource	Reference or source	Identifier or catalog number
	This study	N/A
**Experimental models**		
B16F10	Gift from Dr. Dan Lu’s laboratory	N/A
SF9 insect cell	Invitrogen	Cat#12659017
FreeStyle 293F Cells	Thermo Fisher Scientific	Cat#R79007
C57BL/6JNifdc	Charles River	C57BL∕6JNifdc
**Recombinant DNA**		
pBacMam-hLAT4-GFP-His8-strep	This study	N/A
pBacMam- hLAT4-HA-GFP-His8-strep	This study	N/A
pKSM- hLAT4	This study	N/A
**Antibodies**		
Mouse monoclonal PE anti-HA.11 Epitope Tag Antibody	BioLegend	Cat#901518
**Oligonucleotides and other sequence-based reagents**		
PBM-LAT4 Fwd	5´-caattacagctcttaaggaattcgccaccATGGCGCCCACCCTGGCCACTG-3´	LAT4 full length
PBM-LAT4 Rev	5´-cctccctcgacacctccctcgagCACGAAGGCCTCCTGGTTGGACGAG-3´	LAT4 full length
KSM-LAT4 Fwd	5´-CGCTCAACTTTGGGCCCCTCGAGATGGCGCCCACCCTGGCCACTG-3´	KSM- LAT4 full length
KSM-LAT4 Rev	5´-CCCCCGGGCTGCAGGAATTCttaCACGAAGGCCTCCTGGTTGG-3´	KSM- LAT4 full length
Q500A Fwd	5´-CGCTCTTCGCCCTTCTGCAGgcGCCGCTGTTTCTGGCCATGATGGG-3´	LAT4 Q500A mutant
Q500A Rev	5´-CCCATCATGGCCAGAAACAGCGGCgcCTGCAGAAGGGCGAAGAGCG-3´	LAT4 Q500A mutant
F92S Fwd	5´-CGAGATGCTAAATTTGGCCTcCACTGTGGGCTCCTTTCTGCTCAGTG-3´	LAT4 F92S mutant
F92S Rev	5´-CACTGAGCAGAAAGGAGCCCACAGTGgAGGCCAAATTTAGCATCTCG-3´	LAT4 F92S mutant
I339S Fwd	5´-CGTCACGCAGCTGCGGCTCAgCTTCTACATGGGGGCTATGAACAACATCCTCAAG-3´	LAT4 I339S mutant
I339S Rev	5´-CTTGAGGATGTTGTTCATAGCCCCCATGTAGAAGcTGAGCCGCAGCTGCGTGACG-3´	LAT4 I339S mutant
M342S Fwd	5´-CAGCTGCGGCTCATCTTCTACAgcGGGGCTATGAACAACATCCTCAAG-3´	LAT4 M342S mutant
M342S Rev	5´-CTTGAGGATGTTGTTCATAGCCCCgcTGTAGAAGATGAGCCGCAGCTG-3´	LAT4 M342S mutant
L31A Fwd	5´-CCTCCTCTTCTCGGCAGTCCTCgcGGGCTGGGGCTCGCTGCTCATCATG-3´	LAT4 L31A mutant
L31A Rev	5´-CATGATGAGCAGCGAGCCCCAGCCCgcGAGGACTGCCGAGAAGAGGAGG-3´	LAT4 L31A mutant
Y187A Fwd	5´-CCTTGATGATTGGGTCCGCCGCCTCCTCGGCAGTCACCTTTCCAGG-3´	LAT4 Y187A mutant
Y187A Rev	5´-CCTGGAAAGGTGACTGCCGAGGAGGCGGCGGACCCAATCATCAAGG-3´	LAT4 Y187A mutant
H465A Fwd	5´-CAATCGTGCGAGGATTCATCGCCTCCGCTGTCGGGGGCCTGTACGCTG-3´	LAT4 H465A mutant
H465A Rev	5´-CAGCGTACAGGCCCCCGACAGCGGAGGCGATGAATCCTCGCACGATTG-3´	LAT4 H465A mutant
S100A Fwd	5´-CTGTGGGCTCCTTTCTGCTCGCTGCCATCACCCTGCCCCTGGGTATCG-3´	LAT4 S100A mutant
S100A Rev	5´-CGATACCCAGGGGCAGGGTGATGGCAGCGAGCAGAAAGGAGCCCACAG-3´	LAT4 S100A mutant
Q335A Fwd	5´-CACCATGTGCGTCACGGCGCTGCGGCTCATCTTCTACATGGGGG-3´	LAT4 Q335A mutant
Q335A Rev	5´-CCCCCATGTAGAAGATGAGCCGCAGCGCCGTGACGCACATGGTG-3´	LAT4 Q335A mutant
S489A Fwd	5´-CCTCACGGGACTGCAGgCTCTGATCAGCGCGCTCTTCGCCCTTCTGCAG-3´	LAT4 S489A mutant
S489A Rev	5´-CTGCAGAAGGGCGAAGAGCGCGCTGATCAGAGcCTGCAGTCCCGTGAGG-3´	LAT4 S489A mutant
T485A Fwd	5´-CCACCCAGTTCGGCAGCCTCgCGGGACTGCAGTCTCTGATCAGCGCG-3´	LAT4 T485A mutant
T485A Rev	5´-CGCGCTGATCAGAGACTGCAGTCCCGcGAGGCTGCCGAACTGGGTGG-3´	LAT4 T485A mutant
Q488A Fwd	5´-CGGCAGCCTCACGGGACTGgcGTCTCTGATCAGCGCGCTCTTCGCCCTTCTG-3´	LAT4 Q488A mutant
Q488A Rev	5´-CAGAAGGGCGAAGAGCGCGCTGATCAGAGACgcCAGTCCCGTGAGGCTGCCG-3´	LAT4 Q488A mutant
I184A Fwd	5´-CGGTCCACGTTTATTGCCTTGATGgcTGGGTCCTACGCCTCCTCGGCAG-3´	LAT4 I184A mutant
I184A Rev	5´-CTGCCGAGGAGGCGTAGGACCCAgcCATCAAGGCAATAAACGTGGACCG-3´	LAT4 I184A mutant
M159A Fwd	5´-CTCTGAATGGCTTTGGTGGGgcGTGTATGACCTTCACCTCATTAACACTG-3´	LAT4 M159A mutant
M159A Rev	5´-CAGTGTTAATGAGGTGAAGGTCATACACgcCCCACCAAAGCCATTCAGAG-3´	LAT4 M159A mutant
L166A Fwd	5´-CTTTGGTGGGATGTGTATGACCTTCACCTCAgcAACACTGCCCAACATGTTCGGCG-3´	LAT4 L166A mutant
L166A Rev	5´-CGCCGAACATGTTGGGCAGTGTTgcTGAGGTGAAGGTCATACACATCCCACCAAAG-3´	LAT4 L166A mutant
F163A Fwd	5´-CTTTGGTGGGATGTGTATGACCgcCACCTCATTAACACTGCCCAACATG-3´	LAT4 F163A mutant
F163A Rev	5´-CATGTTGGGCAGTGTTAATGAGGTGgcGGTCATACACATCCCACCAAAG-3´	LAT4 F163A mutant
N89A Fwd	5´-CCAGGCCCAGGACGAGATGCTAgcTTTGGCCTTCACTGTGGGCTCCTTTCTG-3´	LAT4 N89A mutant
N89A Rev	5´-CAGAAAGGAGCCCACAGTGAAGGCCAAAgcTAGCATCTCGTCCTGGGCCTGG-3´	LAT4 N89A mutant
N346A Fwd	5´-CTTCTACATGGGGGCTATGgcCAACATCCTCAAGTTCCTGGTCAG-3´	LAT4 N346A mutant
N346A Rev	5´-CTGACCAGGAACTTGAGGATGTTGgcCATAGCCCCCATGTAGAAG-3´	LAT4 N346A mutant
Q499A Fwd	5´-CAGCGCGCTCTTCGCCCTTCTGgcGCAGCCGCTGTTTCTGGCCATG-3´	LAT4 Q499A mutant
Q499A Rev	5´-CATGGCCAGAAACAGCGGCTGCgcCAGAAGGGCGAAGAGCGCGCTG-3´	LAT4 Q499A mutant
M342A Fwd	5´-CAGCTGCGGCTCATCTTCTACgcGGGGGCTATGAACAACATCCTCAAG-3´	LAT4 M342A mutant
M342A Rev	5´-CTTGAGGATGTTGTTCATAGCCCCCgcGTAGAAGATGAGCCGCAGCTG-3´	LAT4 M342A mutant
S483G Fwd	5´-CCCCTCCACCCAGTTCGGCgGCCTCACGGGACTGCAGTCTCTGATCAGCGCG-3´	LAT4 S483G mutant
S483G Rev	5´-CGCGCTGATCAGAGACTGCAGTCCCGTGAGGCcGCCGAACTGGGTGGAGGGG-3´	LAT4 S483G mutant
D386G Fwd	5´-CATTGGCTACATCATGGgCTGGAGGCTGAAGGAGTGTGAAGACGCCTCCG-3´	LAT4 D386G mutant
D386G Rev	5´-CGGAGGCGTCTTCACACTCCTTCAGCCTCCAGcCCATGATGTAGCCAATG-3´	LAT4 D386G mutant
T178G Fwd	5´-CATGTTCGGCGACCTTCGGTCCggGTTTATTGCCTTGATGATTGGGTCCTACG-3´	LAT4 T178G mutant
T178G Rev	5´-CGTAGGACCCAATCATCAAGGCAATAAACccGGACCGAAGGTCGCCGAACATG-3´	LAT4 T178G mutant
N170G Fwd	5´-CCTTCACCTCATTAACACTGCCCggCATGTTCGGCGACCTTCGGTCCACG-3´	LAT4 N170G mutant
N170G Rev	5´-CGTGGACCGAAGGTCGCCGAACATGccGGGCAGTGTTAATGAGGTGAAGG-3´	LAT4 N170G mutant
S478G Fwd	5´-CCTGTACGCTGCCGTGTACCCCggCACCCAGTTCGGCAGCCTCACGGGACTG-3´	LAT4 S478G mutant
S478G Rev	5´-CAGTCCCGTGAGGCTGCCGAACTGGGTGccGGGGTACACGGCAGCGTACAGG-3´	LAT4 S478G mutant
I180A Fwd	5´-CATGTTCGGCGACCTTCGGTCCACGTTTgcTGCCTTGATGATTGGGTCCTACG-3´	LAT4 I180A mutant
I180A Rev	5´-CGTAGGACCCAATCATCAAGGCAgcAAACGTGGACCGAAGGTCGCCGAACATG-3´	LAT4 I180A mutant
F481A Fwd	5´-CCGTGTACCCCTCCACCCAGgcCGGCAGCCTCACGGGACTGCAG-3´	LAT4 F481A mutant
F481A Rev	5´-CTGCAGTCCCGTGAGGCTGCCGgcCTGGGTGGAGGGGTACACGG-3´	LAT4 F481A mutant
**Chemicals, Enzymes and other reagents**		
Lauryl Maltose Neopentyl Glycol (LMNG)	Anatrace	Cat#NG310
Cholesteryl Hemisuccinate Tris Salt (CHS)	Anatrace	Cat#CH210
SIM SF Insect SF9/SF21 Cell Medium	Sino Biological Inc.	Cat#MSF1
FreeStyle 293 Expression Medium	Gibco	Cat#12338026
LEIBOVITZ L 15 MED	Gibco	Cat#11415064
DMEM basic(1X)	Gibco	Cat#C11995500CP
METHIONINE, L-[METHYL-^3^H]-	American Radiolabeled Chemical	Cat# NETO61X001MC
Methionine	Sigma	M9625-5G
Phenylalanine	Sigma	78019-25 G
L-Isoleucine	MCE	HY-N0771
L-Valine	MCE	HY-N0717
L-Histidine	MCE	HY-N0832
L-Glutamic acid	MCE	HY-14608
L-Threonine	MCE	HY-N0658
L-Proline	MCE	HY-Y0252
L-Alanine	MCE	HY-N0229
Tubeimoside I	MCE	HY-N0890
Timosaponin BII	MCE	HY-N0812
Timosaponin A1	MCE	HY-N6079
Timosaponin AIII	MCE	HY-N0810
Ouabain	MCE	HY-B0542
Gypenoside XLVI	MCE	HY-N6252
Digitonin	Sigma	D5628-1G
**Software**		
CryoSPARC	https://cryosparc.com/	
PyMOL	https://pymol.org/2/	
EPU-2.9.0.1519REL	Thermo Fisher Scientific	N/A
UCSF Chimera	https://www.cgl.ucsf.edu/chimera	
UCSF ChimeraX	https://www.cgl.ucsf.edu/chimerax/	
Coot	https://www.mrc-lmb.cam.ac.uk/personal/pemsley/coot	
Phenix	https://www.phenix-online.org/	
PRISM 8.0 software	https://www.graphpad.com/scientific-software/prism/	
FlowJo v10.6.2	https://www.flowjo.com/	
GROMACS	https://www.gromacs.org/	
**Other**		
Ni-NTA Beads 6FF	LABLEAD	Cat#N30210-100 ml
MagStrep “type3” Strep-Tactin® beads	IBA Lifesciences	2-1613-002
Superose 6 Increase 10/300 GL	GE Healthcare	Cat#29091596
GF-0.6/1.0 300 Au-flat	Electron Microscopy Sciences	Cat# GF-0.6/1.0-3Au-45nm-50

### Cell lines

FreeStyle 293F (Thermo Fisher Scientific) suspension cells were cultured in 293 Expression Medium (Gibco) supplemented with 1% FBS at 37 °C, in an atmosphere of 6% CO_2_ and 60% humidity. B16F10 cells were cultured in DMEM basic (Thermo Fisher Scientific) supplemented with 10% FBS at 37 °C, with 6% CO_2_ and 60% humidity. Sf9 insect cells (Thermo Fisher Scientific) were cultured in Sf-900 III SFM medium (Thermo Fisher Scientific) at 27 °C. The cell lines were routinely checked to be negative for mycoplasma contamination.

### Oocyte expression systems and flux experiments

*Xenopus laevis* oocytes were isolated and maintained as previously described (Plautz et al, 2016). Briefly, ovarian fragments were isolated from the animal and digested with collagenase (2 mg/ml, type IA, Sigma-Aldrich) for 2 h in OR2 (Oocyte Ringer 2) buffer containing 82.5 mM NaCl, 2 mM KCl, 1 mM MgCl_2_ and 5 mM HEPES (pH 7.4). Oocytes were then washed in ND96 buffer containing 96 mM NaCl, 2 mM KCl, 1.8 mM CaCl_2_, 1 mM MgCl_2_, and 5 mM HEPES (pH 7.8). Stage VI oocytes were transferred to fresh OCM containing 60% Leibovitz L15 medium (Thermo Fisher Scientific), 100 μg/ml gentamycin and 10 mM HEPES (pH 7.8) and were maintained at 18 °C prior to injection. cDNAs encoding wild-type and mutant hLAT4 were transferred into the pKSM vector. cRNA was obtained using a T3 mMessage mMachine Kit (Thermo Fisher Scientific) and 25 ng injected into selected oocytes. Oocytes were incubated at 20 °C for 40–48 h in ND96 solution with 100 μg/ml gentamycin to allow expression of LAT4. Uptake was measured in ND96 medium containing the desired amino acid concentration (Thermo Fisher Scientific) and 10 μCi/ml [^3^H]methionine (NET061X, PerkinElmer). For assessment of mutant LAT4 activity relative to wild-type LAT4 as well as the inhibitory effects of substrates and drugs, oocytes were incubated in buffer containing 0.5 mM methionine (10 μCi/ml) for 10 min. Oocytes were solubilized by adding 1% Triton, followed by liquid scintillation fluid to quantify radioactivity. LAT4 activity was calculated by subtracting the uptake by the non-injected control samples from that of the cRNA-injected samples.

### Expression and purification of LAT4

cDNA encoding human wild-type LAT4 was cloned into the BacMam expression vector with C-terminal GFP-His-Strep tags (Li et al, 2017). *E. coli* DH5α and DH10Bac strains were used for plasmid construction. The baculoviruses were produced using the Bac-to-Bac system and amplified in Sf9 cells. For protein expression, HEK293F cells cultured in Freestyle 293 medium at a density of 2.5 × 10^6^/ml were infected with a 10% volume of virus. Sodium butyrate (10 mM) was added to the culture 8 h post-infection, and cells were harvested 60 h post-infection. Cells were collected by centrifugation at 4000 × *g* for 15 min and washed with 20 mM HEPES (pH 7.4) containing 150 mM NaCl.

Cell pellets were solubilized in buffer containing 150 mM NaCl, 20 mM HEPES at pH 7.4 (HBS), 1% (w/v) LMNG (Anatrace), and 0.1% (w/v) CHS (Anatrace) at 4 °C for 1 h. Insoluble components were removed by centrifugation at 70,000 × *g* for 30 min. The supernatant was supplemented with 10 mM imidazole (Sigma-Aldrich) and loaded onto a 5 ml Ni-NTA column (GE Healthcare). Beads were first washed with HBS containing 50 μM glyco-diosgenin (GDN) (Anatrace) and then with HBS containing 50 μM GDN and 20 mM imidazole. Proteins were eluted using HBS with 50 μM GDN and 250 mM imidazole, and tags were removed using TEV protease. For purification of LAT4_DGT_, the eluate was subjected to SEC using a Superose 6 Increase 10/300 GL column (GE Healthcare) in buffer containing 20 mM HEPES, pH 7.4, 150 mM NaCl, and 0.1% (w/v) digitonin (Sigma-Aldrich). For purification of LAT4 in different states, the eluate was used for nanodisc reconstitution. Purified LAT4 was mixed with the circularizable scaffold protein NW9, purified as previously described (Nasr et al, 2017), and lipid mix (POPC:POPE:POPG = 3:1:1) at a molar ratio of 1:2:100 and incubated on ice for 1 h. Detergents were removed by incubation with Biobeads SM2 (Bio-Rad) at 4 °C overnight. Excess NW9 proteins were removed by Strep Tactin^TM^ beads in HBS buffer. The eluate was applied to a Superose 6 Increase 10/300 GL column in HBS buffer. The eluted peak corresponding to LAT4 was collected for cryo-EM sample preparation.

### Cryo-EM sample preparation and data acquisition

Nanodiscs containing LAT4_APO_ and LAT4_PHE_ (the latter supplemented with 10 mM phenylalanine) were diluted to 0.2 mg/ml, and aliquots (4 μl) were applied to reduced graphene oxide-coated grids (Quantifoil Au 300 mesh R1.2/1.3) (Liu et al, 2021). LAT4_DGT_ protein samples were concentrated to 6 mg/ml and aliquots (3 μl) applied to R1.2/1.3 300 mesh Au holey carbon grids (Quantifoil) or GF-0.6/1.0 300 Au-flat (Electron Microscopy Sciences). The cryo-EM grids were prepared using Thermo Fisher Vitrobot Mark IV at 8 °C and 100% humidity (Thermo Fisher Scientific). Grids were screened, and data were collected using a Titan Krios microscope (Thermo Fisher Scientific) at 300 kV for data acquisition. Images were collected using a K3 camera (Gatan) mounted post a Quantum energy filter with a 20 eV slit and operated in super-resolution mode with a pixel size of 0.834 Å at the object plane. Defocus values were set to range from −1.5 μm to −2.0 μm for data collection. Data were acquired using EPU-2.9.0.1519REL. The dose rate on the detector was 15.0 e^−^s^−1^A^−2^ with a total exposure of 56 e^−^A^–2^. Each 2.56 s movie was dose-fractioned into 32 frames.

### Image processing

CryoSPARC software was used for structural analysis (Punjani et al, 2017). The original movies were gain-corrected, motion-corrected and binned to a pixel size of 0.834 Å in a Patch motion correction step. Dose-weighted micrographs were used for CTF estimation using Patch-CTF in cryoSPARC. Particles were auto-picked using Topaz-0.2.3 (Bepler et al, 2019), extracted with a pixel size of 3.336 Å and subjected to two-dimensional (2D) classification to remove contaminants. Particles showing obvious secondary structure features were re-extracted with a pixel size of 1.668 Å and subject to several rounds of ab-initio reconstructions and heterogeneous refinement. Seed-facilitated 3D classification was performed to improve the resolution (Wang et al, 2021). Final particles were re-extracted with a pixel size of 0.834 Å. Non-uniform and local refinement were performed, and resolutions were estimated by gold-standard Fourier shell correlation. A detailed flowchart of the data processing protocol is presented in Appendix Figs. S2–S4.

### Model building

Alphafold (Jumper et al, 2021) was used to build initial models of LAT4 and docked onto the cryo-EM map with UCSF Chimera-1.14 (Pettersen et al, 2004). Models were manually rebuilt in Coot-0.9.2 (Emsley et al, 2010) and further refined by Phenix1.19.2-4158 (Adams et al, 2010). CIF files for ligands were generated in Phenix using eLBOW. The residues contained in the final models are indicated in the Appendix Table. S1. Figures were prepared with Pymol-1.7.0.5 (Schrodinger, LLC) and UCSF ChimeraX-1.5 (Emsley et al, 2010).

### Surface labeling

293F cells were transfected with wild-type and mutant hLAT4 constructs containing an HA tag inserted between G62 and G63 as previously described (Lu et al, 2023). Cells were collected 24 h post-transfection and incubated with PE-conjugated anti-HA tag antibody (BioLegend, cat# 901518) for 30 min at room temperature, followed by analysis by flow cytometry (BD LSRFortessa^TM^) using FlowJo v10.6.2 software.

### ^3^H-labeled methionine uptake assays

Live cell amino acid uptake assays using B16F10 cells were carried out in 48-well plates precoated with poly-D-lysine. Cells were plated at 70% confluency, then washed twice with 200 µl assay buffer (100 mM NaCl, 2 mM KCl, 1 mM CaCl_2_, 1 mM MgCl_2_, and 10 mM HEPES at pH 7.4) to remove cell media. [^3^H]methionine (10 μCi/ml, 0.5 mM) in the same buffer was added to the cells at the same time as TBM-1 (MedChemExpress) and BCH (MedChemExpress) and incubated for 10 min at room temperature. To remove the [^3^H]methionine and inhibitor, cells were washed three times with 200 µl assay buffer and then lysed by adding 1% Triton X-100. Liquid scintillation fluid was then added to allow quantitation of the radioactivity in the cells, which was expressed relative to the protein concentration of the lysates. Protein concentrations of the lysates were determined using a BCA kit according to the manufacturer’s instructions (Thermo Fisher Scientific).

### MD simulations

Simulation systems of LAT4 with phenylalanine, methionine and digitonin were prepared using CHARMM-GUI (Jo et al, 2008). The configurations of digitonin and Tubeimoside-1 were optimized at the B3LYP/6-31 G* level using the Gaussian 09 package. To achieve more realistic stimulations, we used COOT (Emsley et al, 2010) to build the missing flexible regions of the cryo-EM structures. The complex was embedded in a POPC and cholesterol bilayer (10:1 POPC: cholesterol) to generate a suitable membrane system with 22.5 Å layers of water on both sides of the membrane and 150 mM NaCl using CHARMM-GUI.

MD simulations were performed with GROMACS (Salomon-Ferrer et al, 2013) using charmm36m, Lipid14 force field, and generalized Amber force field (GAFF). The topological parameters of digitonin were generated using RESP charge fitting in Antechamber. The particle mesh Ewald (PME) algorithm (Hou et al, 2011) was employed to compute long-range electrostatic energies, and van der Waals and Coulomb interactions were truncated at 10 Å. All hydrogen-related covalent bonds were constrained using the SHAKE algorithm. The system first underwent minimization of protein and ligand side chains. Unfavorable contacts were then removed by 2500 steps of steepest-descent and 2500 steps of conjugate-gradient minimization. The system was gradually heated from 0 to 298 K, and then equilibrated for 500 ps at 1 atm in an isochoric/isothermal (NPT) ensemble with periodic boundary conditions. Temperature and pressure controls were achieved by Nosé-Hoover thermostat and Berendsen barostat, respectively, with a frequency of 2.0 ps. Finally, the equilibrated systems were subjected to 200-ns-long production. All MD simulation processes were performed independently three times. Visualization of the MD trajectories was performed in VMD (Humphrey et al, 1996).

### Molecular docking

To find inhibitors of LAT4, we used the LAT4_DGT_ model without digitonin as a template. Glide of the Schrödinger suite was used for molecular docking (Friesner et al, 2004). LAT4 was prepared with the Protein Preparation Wizard under default parameters (Sastry et al, 2013). The binding site was defined by generating a grid with the Receptor Grid Generation Panel. The binding site outlining box was defined around the reference digitonin ligand in the LAT4 template structure. Compound structures were obtained from PubChem and was prepared for docking using LigPrep with the default parameters, where the possible states were generated at target pH values of 7 ± 2. Compounds with lower Glide scores were considered as candidates for the subsequent functional experiments.

### Mice

Animals used in this study are C57BL/6J mice purchased from Charles River. Female MMTV-PyVT mice were used for the spontaneous breast cancer model and TBM-1 treatment experiments. Animal protocols used for these experiments were approved by the Institutional Animal Care and Use Committee (IACUC) of Peking University Health Science Center (approval number: DLASBE0127). Mice were maintained in a specific pathogen-free animal facility on a 12-h light/12-h dark cycle with free access to water and food, at an ambient temperature of 21 ± 2 °C.

### Single-cell RNA sequencing library preparation and gene expression matrix generation

Breast cancer tissues from MMTV-PyVT mice were dissociated using the Tumor Dissociation Kit (Miltenyi Biotec, 130-096-730). Red blood cells and dead cells were removed using the red blood cell lysis solution (Miltenyi Biotec, 130-094-183) and the dead cell removal kit (Miltenyi Biotec, 130-090-101), respectively. For single-cell sequencing, we utilized a SeekGene SeekOne DD single-cell 3’ transcriptome kit according to the manufacturer’s instructions. Briefly, 13,000 cells of each sample (one for DMSO control and one for TBM-1-treated tissues) were mixed with a single-cell mix, followed by loading onto the chip with barcoded beads and carrier oil. Libraries were generated according to the manufacturer’s instructions and sequenced at 150 paired-end cycles using the Novaseq 6000 system (Illumina). Sequencing bcl2 files were converted into FASTQ files using the SeekSoul tools provided by SeekGene. Sequences were mapped to the USCS hg38 human genome using STAR. A unique molecular identifier (UMI) count matrix was generated and analyzed by the R package Seurat (5.1.0). Cell doublets or multiplets were identified and removed by the Scrublet package (1.0), and only high-quality reads (200 < nCount <8500, nFeature <80,000, percent.mt <20%) were reserved for subsequent analysis. 6769 cells (DMSO control) and 9031 cells (TBM-1 treated) were finally retained, and anchor-based RPCA integration was used for integrating the two samples for follow-up analysis.

### Unsupervised clustering and cell type annotation

Data were normalized and scaled, followed by the identification of highly variable features according to the Seurat vignettes. The FindCluster() function was used with a resolution of 0.1 to classify the cells into ten major clusters. Using the FindAllMarkers() function in Seurat, the most highly expressed genes of each cluster were selected and compared with published references (Reed et al, 2024; Wu et al, 2021) to perform the cell type annotation.

### Identification of malignant cells

To identify the malignant cells within the epithelial cells, the infercnv (1.20.0) package was used to analyze the CNV levels based on mRNA expression levels, using the clusters of immune cells and stromal cells as reference groups with the cutoff set as “0.1” and the “HMM” set as “FALSE”.

### Gene ontology (GO) enrichment analysis

GO enrichment analysis was performed using the clusterProfiler package (4.12.6). Briefly, differentially expressed genes were identified using the FindMarkers() function in the Seurat package. The upregulated gene set and downregulated gene set were then used separately to calculate positively enriched and negatively enriched GO pathways, respectively, with the pAdjustMethod set as “BH” and *p* value and *q* value cutoffs both set as 0.05.

### Quantification and statistical analysis

Global resolution estimations of cryo-EM density maps are based on the 0.143 Fourier Shell Correlation criterion (Rosenthal and Henderson, 2003). The local resolution map was calculated using cryoSPARC-3.1.0 (Punjani et al, 2017). Prism GraphPad software v9.3.0 was used for statistical analysis. The number of biological replicates (*N*) and the relevant statistical parameters for each set of experiments (such as mean or standard error) are described in the figure legends. *P* values were calculated by two-tailed unpaired *t*-tests. Randomization was used to allocate animals to different treatment groups. Samples with technical failure or poor data quality were excluded from the analysis. No statistical methods were used to pre-determine sample sizes.

## Supplementary information

Appendix

Peer Review File

Source data Fig. 1

Source data Fig. 2

Source data Fig. 3

Source data Fig. 4

Source data Fig. 5

Source data Fig. 6

Appendix Figure Source Data

## Data Availability

Cryo-EM maps of hLAT4_APO_, hLAT4_PHE_, hLAT4_DGT_, and hENBT1_APO_ have been deposited in the Electron Microscopy Data Bank under accession codes EMD-61324, EMD-61325, EMD-61326, and EMD-62783, respectively. Atomic models of hLAT4_APO_, hLAT4_PHE_, hLAT4_DGT_, and hENBT1_APO_ have been deposited at the PDB under accession codes 9JBS, 9JBT, 9JBU, and 9L38, respectively. The single-cell RNA sequencing data generated in this study have been deposited in the National Genomics Data Center (NGDC) under the accession code BioProject: PRJCA038601. These data were publicly accessible at https://www.cncb.ac.cn without restrictions. The source data of this paper are collected in the following database record: biostudies:S-SCDT-10_1038-S44318-026-00786-0.
