# Supplementary material for: Structures of the neutral amino acid transporter LAT4 provide insights into antitumor effects of its inhibitor tubeimoside-1
Source: EMBO J. 2026 May 4;45(12):4283–98. doi: 10.1038/s44318-026-00786-0 (PMC13269557; doi:10.1038/s44318-026-00786-0)
Supplement: Supplementary file 1 — Appendix [file 44318_2026_786_MOESM1_ESM.pdf]

Appendix

Structures of the neutral amino acid transporter LAT4 provide insights into antitumor effects of its inhibitor tubeimoside-1

Table of Contents:

Appendix Figure S1 Page 2

Appendix Figure S2 Page 3

Appendix Figure S3 Page 4

Appendix Figure S4 Page 5

Appendix Figure S5 Page 6

Appendix Figure S6 Page 7

Appendix Table S1 Page 8

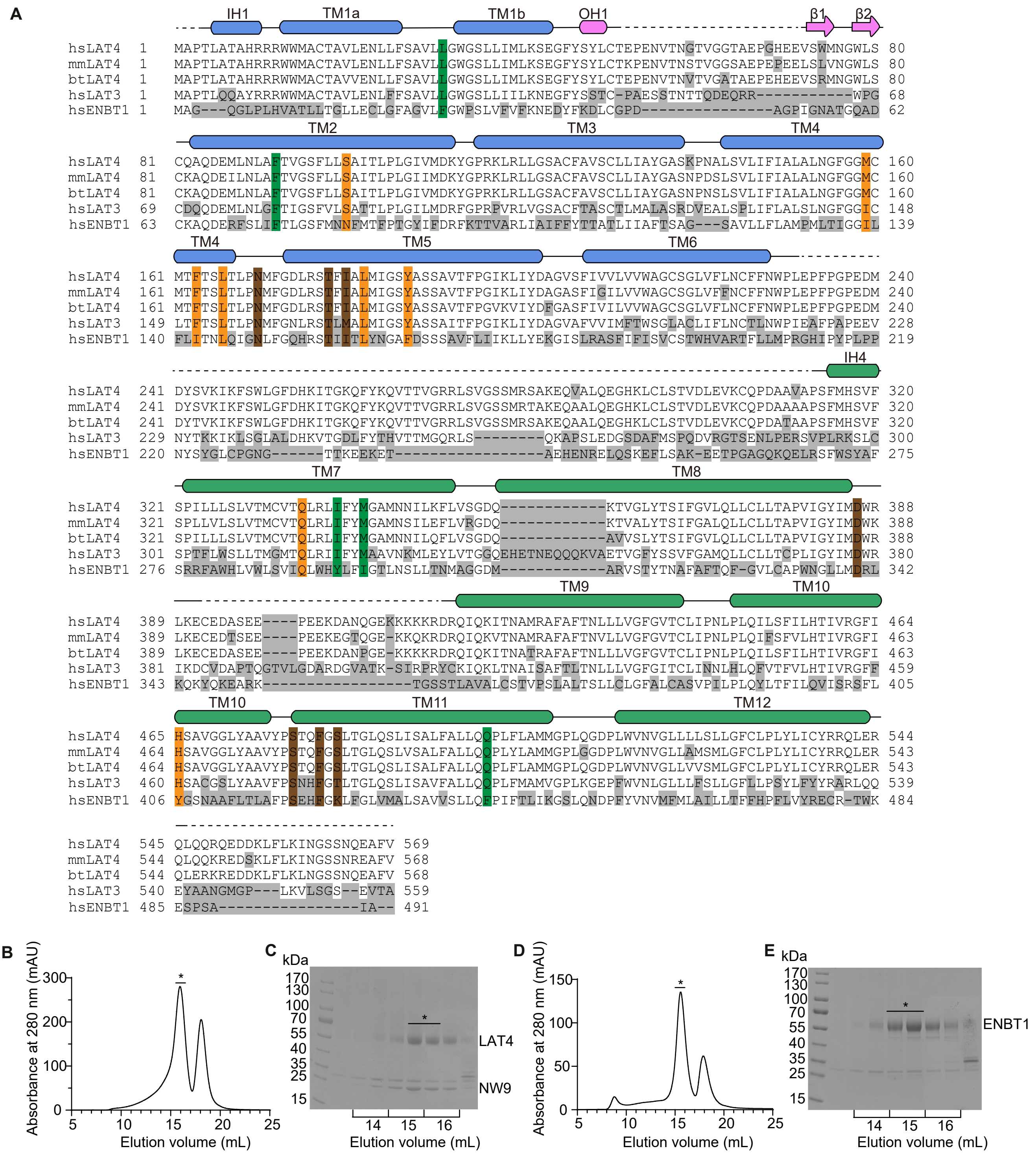

Appendix Figure S1. Sequence alignment and purification of LAT4 and ENBT1.

(A) Sequence alignment of Homo sapiens LAT4 (SLC43A2), Mus musculus LAT4, Bos taurus LAT4, Homo sapiens LAT3 (SLC43A1) and Homo sapiens ENBT1 (SLC43A3). Secondary structures of the LAT4 orthologs are shown above the alignment with the N-terminal lobe, extracellular domain and C-terminal lobe colored in blue, pink and green, respectively. Unmodeled residues are shown as dashed lines. Residues involved in inward and outward structural transitions and substrate coordination are highlighted in brown, green and orange, respectively. Non-conserved residues are highlighted in gray. Alignment was made using PROMALS3D.

(B, C) Size-exclusion chromatography profile (B) and representative SDS-PAGE analysis (C) of purified hLAT4 reconstituted in nanodiscs. The asterisk indicates the fractions used for cryo-EM sample preparation.

(D, E) Size-exclusion chromatography profile (D) and representative SDS-PAGE analysis (E) of purified hENBT1. The asterisk indicates the fractions used for cryo-EM sample preparation.

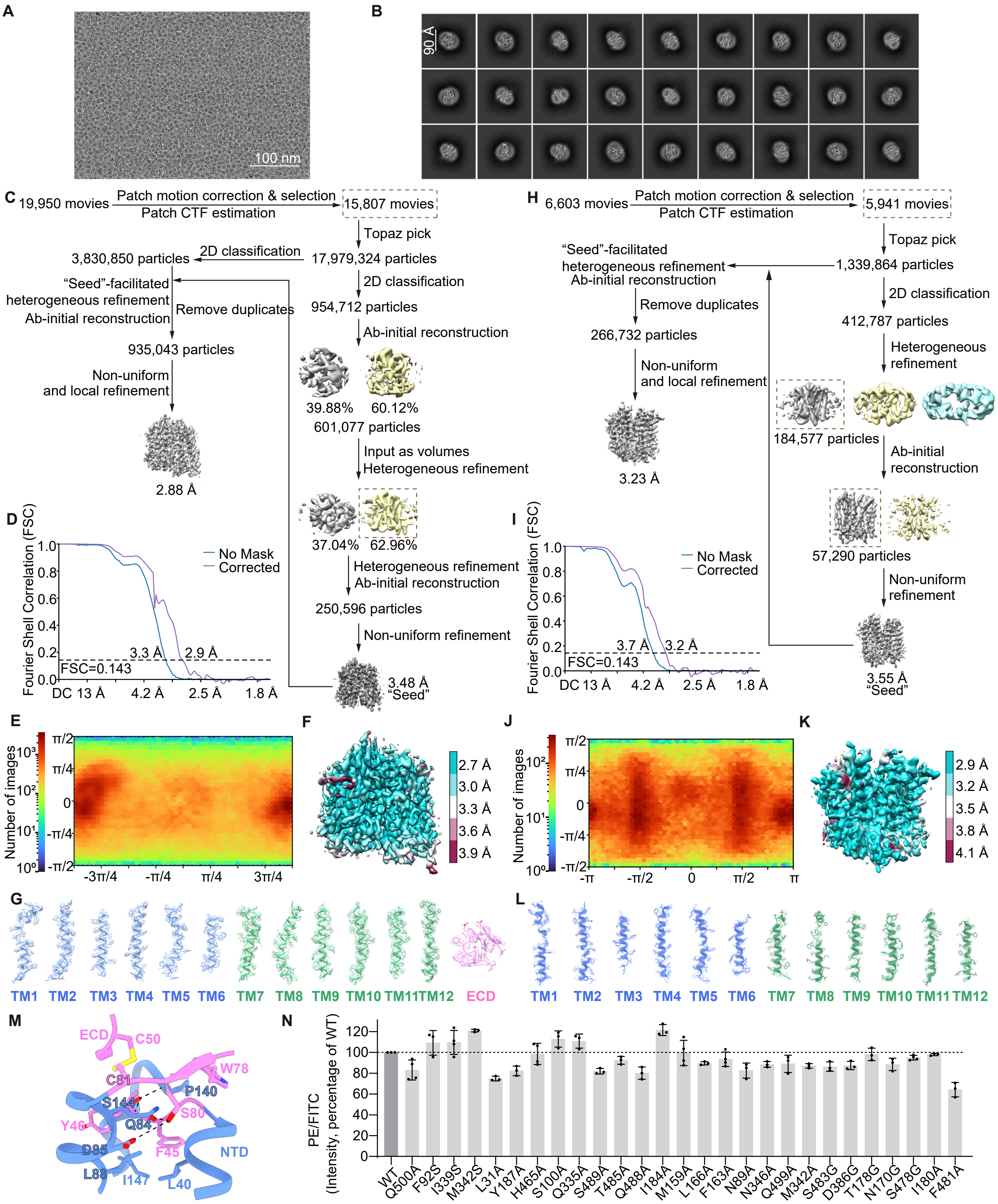

Appendix Figure S2. Cryo-EM image analysis of LAT4 and ENBT1 in the apo state.

(A) Representative raw electron micrograph (19,950 in total) of LAT4 in the apo state (hLAT4$_{APO}$). Scale bar, 100 nm.

(B) Two-dimensional class averages of hLAT4$_{APO}$ output from cryoSPARC. Scale bar, 90 Å.

(C) Flow chart showing the data processing protocols for hLAT4$_{APO}$.

(D) Resolution estimation of hLAT4$_{APO}$ based on the criterion of the FSC 0.143 cut-off.

(E) Angular distribution of the hLAT4$_{APO}$ final reconstruction.

(F) Local resolution distribution of hLAT4$_{APO}$.

(G) Cryo-EM density corresponding to TMs 1-12 and the ECD of hLAT4$_{APO}$ contoured at 9.0 σ.

(H) Flow chart showing the data processing protocols for hENBT1$_{APO}$.

(I) Resolution estimation of hENBT1$_{APO}$ based on the criterion of the FSC 0.143 cut-off.

(J) Angular distribution of the hENBT1$_{APO}$ final reconstruction.

(K) Local resolution distribution of hENBT1$_{APO}$.

(L) Cryo-EM density corresponding to TMs 1-12 of hENBT1$_{APO}$ contoured at 11.3 σ.

(M) Shown is the interface of extracellular loop and N- lobe of hLAT4$_{APO}$. Hydrogen bonds are indicated by dashed lines.

(N) Plasma membrane expression levels of wild-type (WT) and mutant hLAT4 proteins. PE/FITC intensities (anti-HA antibody/GFP fluorescence) of LAT4 mutants have been normalized to WT LAT4. Data for the WT and mutant LAT4 proteins shown are mean values ± SD of n ≥ 3 biologically independent experiments.

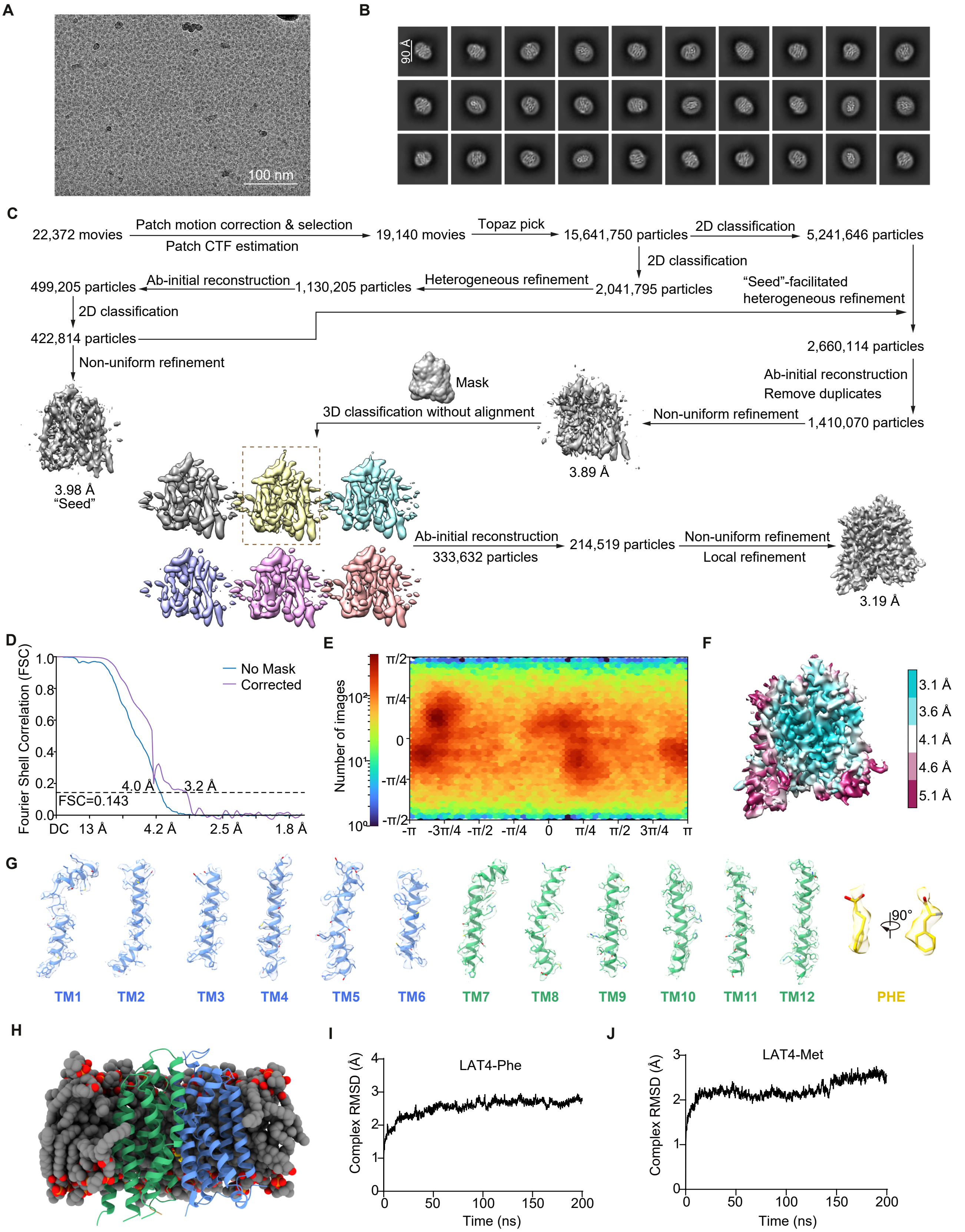

Appendix Figure S3. Cryo-EM image analysis of LAT4 in the phenylalanine-bound state and MD simulations of LAT4 with phenylalanine and methionine.

(A) Representative raw micrograph (22,372 in total) of LAT4 in the phenylalanine-bound state. Scale bar, 100 nm.

(B) Two-dimensional class averages output from cryoSPARC. Scale bar, 90 Å. Similar class averages are observed in Appendix Figure S2B, as both datasets represent LAT4 in the inward-facing state.

(C) Flow chart showing the data processing protocols for $hLAT4_{PHE}$.

(D) Resolution estimation based on the criterion of the FSC 0.143 cut-off.

(E) Angular distribution of the final reconstruction.

(F) Local resolution map of $hLAT4_{PHE}$.

(G) Cryo-EM density corresponding to the TM helices and phenylalanine of $hLAT4_{PHE}$ contoured at 8.6 σ and 6.1 σ (map sharpened with a B factor of -60 Å² in Coot).

(H) Molecular dynamics (MD) system setup for methionine-bound LAT4 embedded in a POPC and cholesterol membrane. LAT4 is shown in cartoon, and the N- and C-lobes are colored in blue and green, respectively. Methionine is shown as sticks and colored in yellow. Lipids are depicted as spheres and colored in gray.

(I, J) Overall structure RMSD of phenylalanine-bound (I) and methionine-bound (J) LAT4 during a 0.2 μs molecular dynamics simulation. The data points were extracted from AmberTools and VMD.

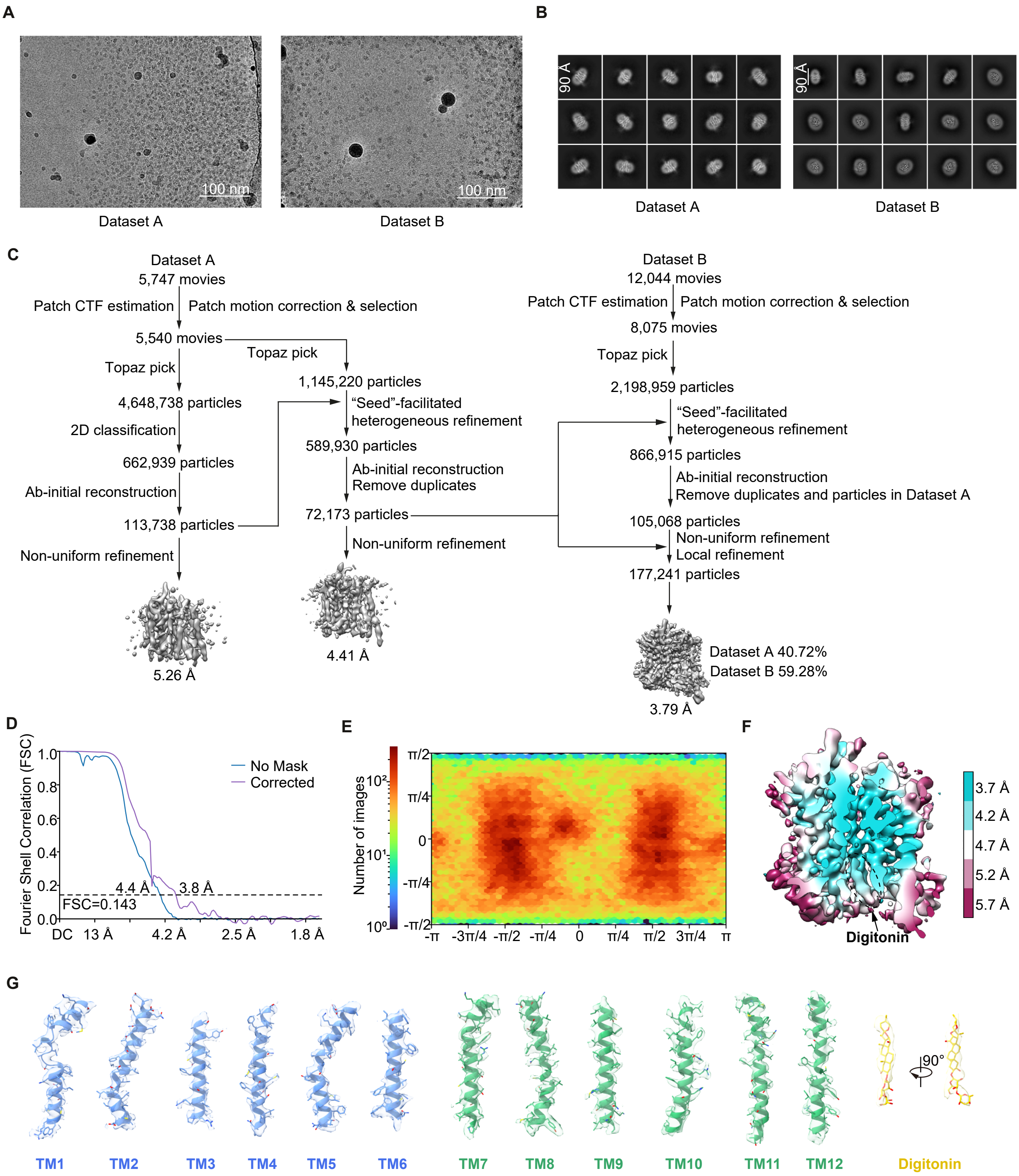

Appendix Figure S4. Cryo-EM image analysis of LAT4 in digitonin-bound state.

(A) Representative raw micrograph (17,791 in total) of LAT4 in a digitonin-bound state. Scale bar, 100 nm.
(B) Two-dimensional class averages output from cryoSPARC. Scale bar, 90 Å.
(C) Flow chart showing the data processing protocols.
(D) Resolution estimation based on the criterion of the FSC 0.143 cut-off.
(E) Angular distribution of the final reconstruction.
(F) A cut-open view of the local resolution map of $hLAT4_{DGT}$. The density corresponding to digitonin is indicated by the arrow.
(G) Cryo-EM densities corresponding to the TM helices and digitonin of $hLAT4_{DGT}$ contoured at 8.5 σ and 5.6 σ.

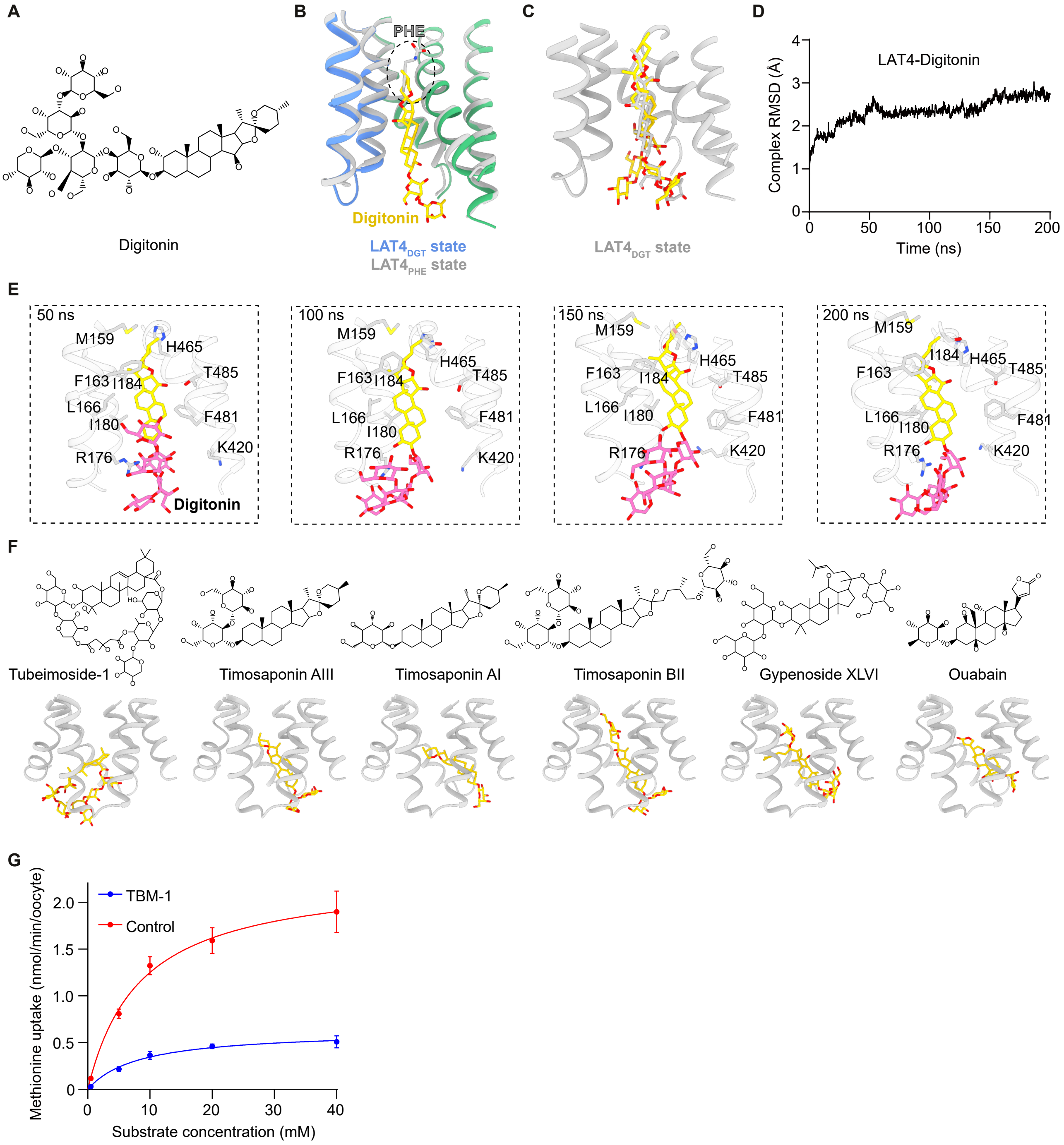

Appendix Figure S5. Molecular docking of digitonin and its analogs used in this study.

(A) Chemical structure of the steroid saponin, digitonin.

(B) Steric clashes between phenylalanine and digitonin are outlined by dashed circles. The N-lobe and C-lobe of $hLAT4_{DGT}$, digitonin and $hLAT4_{PHE}$ are colored in blue, green, yellow and silver, respectively. Phenylalanine and digitonin are shown as sticks.

(C) Docking of digitonin to the hLAT4 structure. $hLAT4_{DGT}$ and docking digitonin (docking scores: -8.68 kcal/mol) are colored in silver and yellow, respectively. Digitonin is shown as sticks.

(D) Overall structure RMSD of digitonin-bound LAT4 during a 0.2 μs MD simulation. The data points were extracted from AmberTools and VMD.

(E) Representative snapshots for MD simulations of digitonin binding to hLAT4 at regular intervals of 50 ns. The digitogenin moiety and the sugars are colored in yellow and pink, respectively.

(F) Docking poses of anti-tumor saponins in the central cavity of LAT4. Docking scores of tubeimoside-1 (TBM-1), timosaponin AIII, timosaponin A1, timosaponin BII, Gypenoside XLVI and ouabain are -6.37, -6.47, -5.25, -9.25, -7.91 and -5.78 kcal/mol, respectively.

(G) Concentration-dependent uptake of methionine in Xenopus oocytes in the absence (Control) or presence of 50 μM TBM-1. Oocytes were incubated in buffer containing [^{3}H]methionine (1 μCi) at the indicated concentrations for 10 min at room temperature. Data points represent mean values ± SEM (n = 3 biologically independent experiments).

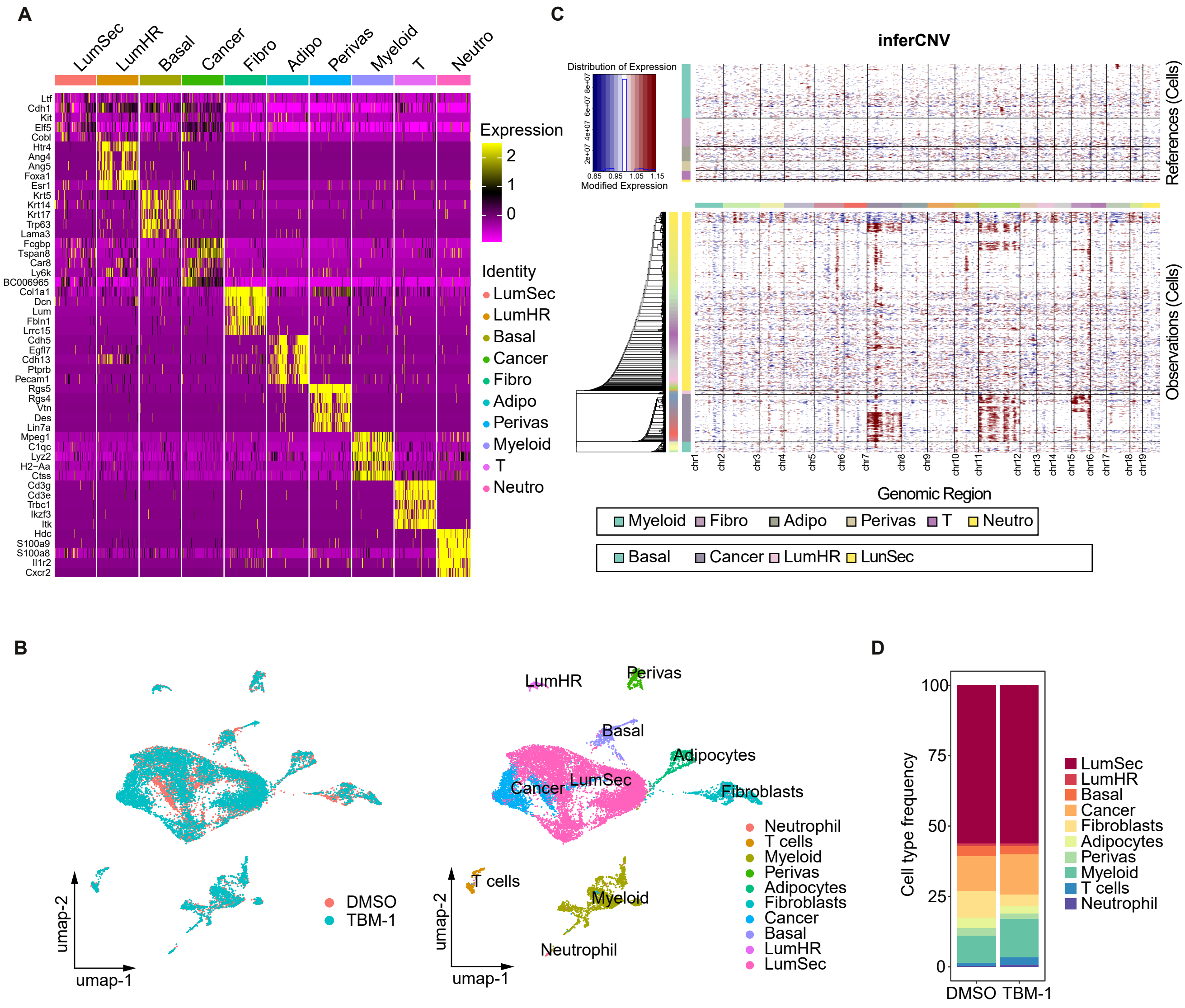

Appendix Figure S6. Inhibition of LAT4 in murine breast cancer cells by TBM-1.
(A) Heatmap showing expression profiles of 5 representative cell-type specific markers of 10 major cell clusters. 50 cells were randomly sampled and shown for each cluster.
(B) Uniform manifold approximation and projection (UMAP) visualization of scRNA-seq data from breast cancer tissues from DMSO (control) and TBM-1 treated mice, colored by original identity (left panel) and by cell types (right panel).
(C) Heatmap showing CNV profiles for individual cells. Non-malignant immune and stromal cells were set as references (top). Large-scale CNVs were observed in the malignant cells (bottom). Red indicates genomic amplification and blue indicates genomic deletions.
(D) Stacked bar plot showing relative proportion of each of the 10 clusters in the DMSO (control) and TBM-1 treated mice.

Appendix Table S1 Cryo-EM data collection, refinement and validation statistics.

	$LAT4_{APO}$	$ENBT1_{APO}$	$LAT4_{PHE}$	$LAT4_{DGT}$
PDB ID	9JBS	9L38	9JBT	9JBU
EMDB ID	61324	62783	61325	61326
**Data collection and processing**				
Magnification	105,000 ×	105,000 ×	105,000 ×	105,000 ×
Voltage (kV)	300	300	300	300
Electron exposure ($e^-/Å^2$)	56	56	56	56
Defocus range (µm)	-1.5 to -2.0	-1.5 to -2.0	-1.5 to -2.0	-1.5 to -2.0
Pixel size (Å)	0.834	0.834	0.834	0.834
Symmetry imposed	*C1*	*C1*	*C1*	*C1*
Initial particle images (no.)	17,979,324	1,339,864	15,641,750	6,847,697
Final particle images (no.)	935,043	266,732	214,519	177,241
Map resolution (Å)	2.9	3.2	3.2	3.8
FSC threshold	0.143	0.143	0.143	0.143
Map resolution range (Å)	250-2.9	250-3.2	250-3.2	250-3.8
**Refinement**				
Initial model used (PDB code)	AF-Q8N370-F1	AF-Q8NBI5-F1	AF-Q8N370-F1	AF-Q8N370-F1
Model resolution (Å)	3.0	3.4	3.9	4.1
FSC threshold	0.5	0.5	0.5	0.5
Model resolution range (Å)	250-3.0	250-3.4	250-3.9	250-4.1
Map sharpening *B* factor ($Å^2$)	-131.8	-121.0	-121.6	-162.5
Model composition				
Non-hydrogen atoms	3,207	3,267	3,101	3,219
Protein residues	415	412	409	415
Ligands			1	1
B factors ($Å^2$)				
Protein	33.47	43.66	72.59	96.58
Ligand			73.66	127.20
R.m.s. deviations				
Bond lengths (Å)	0.004	0.003	0.004	0.002
Bond angles (°)	0.864	0.503	0.884	0.436
Validation				
MolProbity score	1.60	1.63	1.45	1.46
Clashscore	7.65	7.28	5.60	6.17
Poor rotamers (%)	1.71	1.98	1.53	1.46
Ramachandran plot				
Favored (%)	98.28	98.50	98.00	99.26
Allowed (%)	1.72	1.50	2.00	0.74
Disallowed (%)	0.00	0.00	0.00	0.00
