# Supplementary material for: Structures of the neutral amino acid transporter LAT4 provide insights into antitumor effects of its inhibitor tubeimoside-1
Source: EMBO J. 2026 May 4;45(12):4283–98. doi: 10.1038/s44318-026-00786-0 (PMC13269557; doi:10.1038/s44318-026-00786-0)
Supplement: Supplementary file 2 — Peer Review File [file 44318_2026_786_MOESM2_ESM.pdf]

THE EMBO JOURNAL

Structures of the neutral amino acid transporter LAT4 provide insights into antitumor effects of its inhibitor tubeimoside-1

Dian Ding, Yishuo Lu, Jingyi Yang, Hongyi Chen, Peijun Jiang, Yan Jin, Jianyuan Luo, Guangxi Wang, and Yuxin Yin

Corresponding author: Yuxin Yin (yinyuxin@hsc.pku.edu.cn)

Review Timeline:

Submission Date:	18th Aug 25
Editorial Decision:	1st Sep 25
Revision Received:	25th Jan 26
Editorial Decision:	20th Feb 26
Revision Received:	16th Mar 26
Accepted:	27th Mar 26

Editor: Ieva Gailite

Transaction Report:

(Note: With the exception of the correction of typographical or spelling errors that could be a source of ambiguity, letters and reports are not edited. Depending on transfer agreements, referee reports obtained elsewhere may or may not be included in this compilation. Referee reports are anonymous unless the Referee chooses to sign their reports.)

1st Editorial Decision 1st Sep 2025

Dear Dr. Yin,

Thank you for submitting your manuscript for consideration by the EMBO Journal. We have now received comments from three reviewers, which are included below for your information.

As you will see, all reviewers appreciate the findings and their broader novelty. At the same time, they indicate a number of important concerns that need to be addressed before they can support publication here. In particular, reviewer #2 requests a validation of LAT4 and its point mutant surface expression in oocytes. Furthermore, both reviewers #2 and #3 ask for a validation of the dependence of the TBM-1 effect on methionine transport and tumour growth on LAT4 inhibition.

Based on the general interest expressed in the reports, I invite you to submit a revised manuscript in response to the comments by all reviewers, especially focusing on the aspects outlined above. I would be happy to discuss the revision in more detail via email or phone/videoconferencing.

We generally allow three months as standard revision time, which can be extended to six months in the case of major revisions. Should you foresee a problem in meeting this deadline, please let us know in advance to discuss an extension.

As a matter of policy, competing manuscripts published during this period will not negatively impact on our assessment of the conceptual advance presented by your study. However, please contact me as soon as possible upon publication of any related work to discuss the appropriate course of action.

When preparing your letter of response to the referees' comments, please bear in mind that this will form part of the Review Process File and will therefore be available online to the community. For more details on our Transparent Editorial Process, please visit our website: https://www.embopress.org/page/journal/14602075/authorguide#transparentprocess. Please also see the attached instructions for further guidelines on preparation of the revised manuscript.

Please feel free to contact me if you have any further questions regarding the revision. Thank you for the opportunity to consider your work for publication. I look forward to discussing your revision.

Yours sincerely,

Ieva Gailite

Ieva Gailite, PhD
Senior Scientific Editor
The EMBO Journal
Meyerhofstrasse 1
D-69117 Heidelberg
Tel: +4962218891309
i.gailite@embojournal.org

Instructions for preparing your revised manuscript:

Please make sure you upload a letter of response to the referees' comments together with the revised manuscript.

Please also check that the title and abstract of the manuscript are brief, yet explicit, even to non-specialists.

When assembling figures, please refer to our figure preparation guideline in order to ensure proper formatting and readability in print as well as on screen:
https://bit.ly/EMBOPressFigurePreparationGuideline
See also guidelines for figure legends: https://www.embopress.org/page/journal/14602075/authorguide#figureformat

At EMBO Press we ask authors to provide source data for the main manuscript figures. You will receive a separate email with instructions for providing source data with your revised manuscript, including how to upload and organize the files.

IMPORTANT: When you send the revision we will require
- a point-by-point response to the referees' comments, with a detailed description of the changes made (as a word file).
- a word file of the manuscript text.
- individual production quality figure files (one file per figure)

- a complete author checklist, which you can download from our author guidelines (https://www.embopress.org/page/journal/14602075/authorguide).
- Expanded View files (replacing Supplementary Information)
Please see out instructions to authors
https://www.embopress.org/page/journal/14602075/authorguide#expandedview
- a Reagents and Tools Table as part of the Methods section, which can be downloaded from our author guidelines (https://www.embopress.org/page/journal/14602075/authorguide#structuredmethods)

Please remember: Digital image enhancement is acceptable practice, as long as it accurately represents the original data and conforms to community standards. If a figure has been subjected to significant electronic manipulation, this must be noted in the figure legend or in the 'Materials and Methods' section. The editors reserve the right to request original versions of figures and the original images that were used to assemble the figure.

Further information is available in our Guide For Authors: https://www.embopress.org/page/journal/14602075/authorguide

We realize that it is difficult to revise to a specific deadline. In the interest of protecting the conceptual advance provided by the work, we recommend a revision within 3 months (30th Nov 2025). Please discuss the revision progress ahead of this time with the editor if you require more time to complete the revisions.

Referee #1:

This study presents the apo and phenylalanine-bound structures of LAT4 and the structure of the related ENBT1 resolved by cryo-electron microscopy. The resolution for a protein of this small size is very good and as a result the details are quite reliable. Extensive site-directed mutagenesis was performed to support the role of individual residues. As admitted by the authors TBM-1 is a rather unspecific compound and its mild antitumor effects may or may not include methionine restriction.
Specific comments:
Line 82-96: Please note that there is significant redundancy of methionine uptake in cancer cells and the reported effects may have secondary causes other than reduction of methionine uptake. It is not an essential gene in cancer cells (https://depmap.org/portal/gene/SLC43A2?tab=overview).
Fig. 2a: Typically, some non-competing amino acids are shown. The transporter is, however, quite well characterised.
Line 156-157: I am not sure whether the orientation and distance of H465 is suitable for a cation-pi interaction. It looks rather like a hydrophobic pocket to me.
Fig. 6a and c. I have difficulties reconciling the difference of the 5 biggest tumors with the bar graph.
Line 304,305: I would object to the statement that LAT4 is the critical transporter for methionine accumulation. This would rather be LAT1 or SNAT2. The authors can verify this using the GTex portal.

Referee #2:

This manuscript describes very relevant CryoEM structures of human LAT4 (APO and with Phe substrate bound) and ENBT1 in inward-facing and outward-facing conformation respectively, which together with functional analysis of relevant residues and Molecular Dynamics sets the first structure/functional information describing the transport mechanism of amino acids by LAT4. All this information is sufficient to warrant publication in Embo J. To this part of the manuscript, I have two major concerns that need attention. It looks that functional transport studies of LAT4 mutants has been performed in Xenopus oocytes, whereas surface expression of these mutants has been assayed in HEK 293F cells. This is bizarre (see below).
In contrast to this shorter vision, the manuscript includes an interesting last part, the identification of Tubeimoside-1 (TBM-1) as an inhibitor of LAT4 with larger potency that the general L system transporter BCH that decreases tumor growth in a breast cancer mouse model. Because the authors also determined a CryoEM structure of LAT4 with digitonin bound (LAT4(DGT) occupying part of the substrate binding side of the hydrophobic side chain of Phe in a position highly suggesting that this saponin would block occlusion of the cytoplasmic gate of the transporter. This prompted authors to test transport inhibition by other saponins, and identified TBM-1 as the stronger inhibitor of the tested molecules. Docking analysis support binding of BMT-1 in the digitonin locus, and functional analysis support the inhibitory role of TBM-1 on LAT4 transport activity; to my knowledge this is the first potent inhibitor of human LAT4. Then, the authors show that TBM-1 compromises Met absorption in mouse, decrease tumor progression, altered expression of Met-metabolism genes in the tumor cells and increased the presence of T cells in the tumor infiltrates (scRNA-seq).This allows the authors to propose that TMB-1 is a potential lead compound to cause Met Restriction to treat cancer due to the Met vulnerability of cancer cells. I like the idea, but I have a major point and two suggestions.

LAT4 Structure/functional relationship and transport mechanism part

1. CONCERN: The authors show the surface expression of the mutants expressed in HEK cells to demonstrate that the studied mutants does not significantly affect proper expression of the mutated version of the transporter whereas the transport activity was assayed in Xenopus oocytes. The authors must justify this protocol. Moreover, the cell surface expression of mutants is shown in Fig. S2n. This figure is not quoted in the text and there is no indication whatsoever in the text that proper expression of the mutants supports the functional role of the relevant identifies residues. Please, direct the reader in the text to this important information. I strongly suggest to the authors to demonstrate that the assayed mutants show no defect expression in Xenopus oocytes. Taking into account that the authors shown proper surface expression in HEK cells, just WB analysis in Xenopus oocytes would support that defective activity of these mutants in oocytes is not due to compromised expression in the expression system used to analyze the transport function of the studied mutants.

2. CONCERN: HEK293F cells were transfected with HA-tagged wild-type and mutant hLAT4 constructs for surface labelling using PE-conjugated anti-HA tag antibody followed by flow cytometry to show proper surface expression of the studied mutants. There is no description of the location of the HA tag. Taking into account that both N- and C-terminal are intracellular, HA tag in the N- or C-term would be not detected by flow cytometry. Please, indicate the location of the HA tag in these studies. In general, it is a bizarre protocol because 3H-Met uptake was studied in Xenopus oocytes without tagged LAT4 and LAT4 surface expression in human cells with a tagged version.

3. Minor point: please, also indicate in the transport figures the concentration of 3H-Met used in these studies. The information is only found in Mat and Met section. Please, make the exercise of reading your paper easier to the readers.

TBM-1 as a potential leading compound to develop a new cancer therapy.

1. CONCERN: The authors, as indicated above, show that TBM-1 inhibits traced 3H-Met uptake in oocytes expressing LAT4 with IC50 about 1000-fold lower that the general L-system transport inhibitor BCH. Also importantly, TBM-1 barely decreases the accumulation of 3H-Met in blood 1 h to 10 h after an oral gavage (from 500 CPM to 400 CPM from a basal of 50 CPM). What it is not shown is that administration of TBM-1 decreases Met concentration in plasma or blood? Surprisingly, the authors support the idea that the small effect of TBM-1 in 3H-Met absorption (gavage to blood) causes Met Restriction that results in the decrease of tumor progression (volume and weight) in MMTV-PyVT mice. I like the idea but the small effect on 3H-Met gavage accumulation in blood seems too small to decrease tumor progression and increase T cells in the tumor infiltrate showed in the manuscript. Plasma murine Met concentration is around 20 uM that can reach 5 uM with Met Restriction conditions. I ask the authors to show if TBM-1 treatment decrease Met concentration in plasma or blood of MMTV-PyVT mice comparable with the Met Restriction protocols affecting tumor growth quoted in the introduction.

2. SUGGESTION: t-he abstract and the introduction highlight the overexpression of LAT4 in several cancer cells with the following scenario (line 81): "Cancer cells with increased LAT4 expression consumed and outcompeted T cells for methionine which in turn impaired T cell immunity by disrupting methionine metabolism, increasing PD-1 expression in tumor-infiltrating CD4+ 83 T cells and decreasing intracellular SAM in CD8+ 84 T cells (Bian et al., 2020, Pandit et al., 2023)". Does MMTV-PyVT tumor cells express LAT4? If these cells do express the transporter what is the direct impact of TBM-1 in these tumor cells? As clearly indicated in the literature quoted in the introduction LAT4 is a Met diffuser (i.e., net transport goes from a compartment of high concentration to another of lower concentration) that play a role in the basolateral efflux of Met (and Phe and Branched neutral amino acids- in agreement with the main proposition of the present manuscript. This transport mechanism differ from the other L system transporters (LAT1 and LAT2) that are exchangers (i.e., tertiary amino acid active transporters). In this scenario, considering that intracellular AA concentrations are larger than in plasma, one is forced to think that LAT4 in cancer cells would facilitate the efflux of Met and the other AA substrates from the tumor cells to the extracellular space. Then, what is the advantage for tumor cells to overexpress LAT4? Could the authors consider this issue in the discussion?

3. SUGGESTION: Allosteric inhibitors have the advantage that do not compete with substrate to inhibit the transporter activity. Calculated TBM-1 binding site partially coincides substrate Phe binding site and also suggest that would block the cytosolic gate. Thus, TBM-1 could be a noncompetitive inhibitor of human LAT4. I suggest the authors to check whether TBM-1 would affect transport Vmax in a way largely independent of the substrate concentration.

Referee #3:

The study by Ding et al. reports the first structures of the methionine transporter LAT4 (SLC43A2) in multiple states, and also the structure of ENBT1 (SLC43A3). Coupled with these structural studies, the authors use mutagenesis to tease apart the substrate binding and transport cycle of the proteins. Finally, the authors identify a saponin binding site in LAT4 and use this as the basis for a screen for inhibitors. They identify tubeimoside-1 (TBM-1) as a potential inhibitor, characterizing the activity of this molecule on methionine transport via cell and mouse studies.

The structural and biochemical aspects of the study are well designed, executed, analyzed and presented. However, the characterization of TBM-1 as a LAT4 inhibitor is incomplete and therefore the interpretability of these results is uncertain. Furthermore, the structure of ENBT1 is included in this study, but the protein is not introduced or discussed in any detail. Therefore, the significance of this structure is unclear (and likely under-exploited), other than providing the outward-facing

structure of a close paralog for analysis of the LAT4 structures.

This manuscript will require significant revision before publication, though ultimately I believe this is achievable. While the authors decision, I strongly encourage removing the mouse studies that are of questionable value, and adding an analysis of ENBT1.

Critical
- The screen for saponins (Fig 5e) requires tests with and without LAT4 for each compound, as these molecules can non-specifically interact with the membrane and may each have unique off-targets.
- The relatively low affinity of TBM-1 for LAT4, and the absence of a screen for off-target proteins (CETSA) and PK/PD data all make the mouse studies very difficult to interpret. It is unclear if LAT4 is the therapeutic target for the in vivo experiments, though this is implied. In fact, the authors note TBM-1 is already known to affect other pathways (Lines 327-329).

Major
- ENBT1 is only mentioned briefly in the abstract, and not noted at all in the introduction. The background of this protein should be discussed, along with its relevance to LAT4.

Minor
- (Line 114) How are nanodiscs meant to help avoid preferred orientation?
- (Lines 172-173) It is odd to assert Phe and Met share a binding pocket when the residues shown in Fig. 3a and 3c are different. Perhaps show an overlay of the substrate bound-structures, combine Fig 3b and 3d, and/or make an equivalent of Fig 3e for the Phe binding.
- (Line 184) The authors assert these mutations alter the substrate permeation pathway. It would be helpful to clearly label these locations on the protein model.
- (Line 201) I am not clear what states are being compared. Is this bound vs apo, or LAT1-IF-apo vs ENBT1-OF-apo?
- (Lines 229-231) The mention of pH sensitive MFS is unexpected and confusing. Has it been shown if LAT4 is (or is not) pH sensitive? This is only relevant if LAT4 has been demonstrated NOT to be pH sensitive, and if pH sensitive this could arise from residues outside this region.
- (Line 246) The authors assert that digitonin binds stably in the MD simulations. However, this does not appear supported by the data. Fig S5d shows an increasing RMSD, while the RMSD for substrates plateaus.
- (Line 388) The lipid mix should be specified.
- How was the Met placed for MD?
- I find Figs 1e, 1f, 2f, 4c, and 4d hard to interpret due to showing the protein surface as empty shell for slice and also the secondary structure. I suggest removing the secondary structure model and visualizing this volume as a slice through a solid object with neutral interior color such as grey.
- It is difficult to see the hydrophobicity of the LAT4 binding site in Fig 2f. I suggest zooming into this region.
- The fraction of particles that are use in the final reconstructions is relatively small. Are the excluded particles "junk" or genuine protein but conformationally heterogenous? And if flexible, did the authors attempt to model this flexibility using available methods?

Point-by-point response to reviewers

We are grateful for the thorough evaluation of our manuscript from the reviewers. A detailed point-by-point response to all comments and suggestions of the reviewers are provided in this document.

Referee #1:

This study presents the apo and phenylalanine-bound structures of LAT4 and the structure of the related ENBT1 resolved by cryo-electron microscopy. The resolution for a protein of this small size is very good and as a result the details are quite reliable. Extensive site-directed mutagenesis was performed to support the role of individual residues. As admitted by the authors TBM-1 is a rather unspecific compound and its mild antitumor effects may or may not include methionine restriction.

We greatly appreciate the valuable information provided, as well as the insightful suggestions and highly constructive, helpful comments.

Specific comments:

Line 82-96: Please note that there is significant redundancy of methionine uptake in cancer cells and the reported effects may have secondary causes other than reduction of methionine uptake. It is not an essential gene in cancer cells (https://depmap.org/portal/gene/SLC43A2?tab=overview).

Response: We appreciate the reviewer's insightful comment regarding the redundancy of methionine uptake in cancer cells and the non-essential nature of SLC43A2. We fully agree that methionine and SLC43A2 may be involved in additional signaling pathways beyond directly supporting cancer cell survival. Indeed, previous reports on the anti-tumor effects of MR or SLC43A2 inhibition consistently highlight the involvement of immune system activation rather than a sole dependence on methionine uptake. In line with these findings, our single-cell RNA-seq analysis and flow cytometry results revealed increased activation of $CD4^{+}$ T cells and M1 macrophages, along with suppression of M2 macrophages, collectively indicating enhanced anti-tumor immunity. Accordingly, we have revised lines 92–99 to better clarify our research rationale and provide more accurate background information as suggested.

Fig. 2a: Typically, some non-competing amino acids are shown. The transporter is, however, quite well characterised.

Response: We appreciate the reviewer's suggestion and have selected representative non-competing amino acids for measurement, using their physicochemical properties (e.g., polarity, hydrophobicity, and side-chain structure) as the basis for selection. These results have been incorporated into Fig. 2A as suggested.

Line 156-157: I am not sure whether the orientation and distance of H465 is suitable for a cation-pi interaction. It looks rather like a hydrophobic pocket to me.

Response: We note that the typical distance for a cation–π interaction between a positively charged group and the center of an aromatic ring is generally within the range of 4–6 Å, which is consistent with a possible cation–π interaction between Phe and H465 in our model. However, considering the observation that the interaction between Met and H465 is more consistent with hydrophobic contacts, and that such hydrophobic interactions may be more prevalent among other LAT4 substrates, we have revised lines 156–158 to indicate hydrophobic interaction as suggested.

Fig. 6a and c. I have difficulties reconciling the difference of the 5 biggest tumors with the bar graph.

Response: The difficulty in reconciling the five largest tumors between Figs. 6A and 6C may have been caused by a lack of clarity in Fig. 6A due to our oversight in labeling the excised tumors with their corresponding mouse IDs. To address this issue, we have now labeled each tumor in Fig. 6A with both the mouse ID and tumor number. This update allows direct correspondence between the individual tumor sizes in Fig. 6A and the quantitative data in Fig. 6B and 6C as suggested.

Line 304,305: I would object to the statement that LAT4 is the critical transporter for methionine accumulation. This would rather be LAT1 or SNAT2. The authors can verify this using the GTex portal.

Response: We thank the reviewer for the valuable comment and for bringing this to our attention. Our previous focus was on the role of LAT4, however, we agree that LAT1 and SNAT2 are also important transporters for methionine (Guetg et al., 2015, PMID:25480797; Mastrototaro et al., 2016, PMID:27753190). We have revised lines 309–310 to provide a more accurate description as suggested.

Referee #2:

This manuscript describes very relevant CryoEM structures of human LAT4 (APO and with Phe substrate bound) and ENBT1 in inward-facing and outward-facing conformation respectively, which together with functional analysis of relevant residues and Molecular Dynamics sets the first structure/functional information describing the transport mechanism of amino acids by LAT4. All this information is sufficient to warrant publication in Embo J. To this part of the manuscript, I have two major concerns that need attention. It looks that functional transport studies of LAT4 mutants has been performed in Xenopus oocytes, whereas surface expression of these mutants has been assayed in HEK 293F cells. This is bizarre (see below).

In contrast to this shorter vision, the manuscript includes an interesting last part, the identification of Tubeimoside-1 (TBM-1) as an inhibitor of LAT4 with larger potency that the general L system transporter BCH that decreases tumor growth in a breast cancer mouse model. Because the authors also determined a CryoEM structure of LAT4 with digitonin bound (LAT4(DGT) occupying part of the substrate binding side of the hydrophobic side chain of Phe in a position highly suggesting that this saponin would block occlusion of the cytoplasmic gate of the transporter. This prompted authors to test transport inhibition by other saponins, and identified TBM-1 as the stronger inhibitor of the tested molecules. Docking analysis support binding of BMT-1 in the digitonin locus, and functional analysis support the inhibitory role of TBM-1 on LAT4 transport activity; to my knowledge this is the first potent inhibitor of human LAT4. Then, the authors show that TBM-1 compromises Met absorption in mouse, decrease tumor progression, altered expression of Met-metabolism genes in the tumor cells and increased the presence of T cells in the tumor infiltrates (scRNA-seq).This allows the authors to propose that TMB-1 is a potential lead compound to cause Met Restriction to treat cancer due to the Met vulnerability of cancer cells. I like the idea, but I have a major point and two suggestions.

We thank this reviewer for the thorough evaluation of our structures and the highly constructive and helpful comments.

LAT4 Structure/functional relationship and transport mechanism part

1. CONCERN: The authors show the surface expression of the mutants expressed in HEK cells to demonstrate that the studied mutants does not significantly affect proper expression of the mutated version of the transporter whereas the transport activity was assayed in Xenopus oocytes. The authors must justify this protocol. Moreover, the cell surface expression of mutants is shown in Fig. S2n. This figure is not quoted in the text and there is no indication whatsoever in the text that proper expression of the mutants supports the functional role of the relevant identifies residues. Please, direct the reader in the text to this important information. I strongly suggest to the authors to demonstrate that the assayed mutants show no defect expression in Xenopus oocytes. Taking into account that the authors shown proper surface expression in HEK cells, just WB analysis in Xenopus oocytes would support that defective activity of these mutants in oocytes is not due to compromised expression in the expression system used to analyze the transport function of the studied mutants.

Response: Thank you for the insightful suggestion. We have cited Appendix Fig. S2N in lines 181-182, as requested, to indicate that the mutants are properly expressed at the cell surface

and to facilitate access to this information for the reader. We initially chose the 293F expression system to investigate whether mutations introduced into LAT4 result in protein misfolding and decreased membrane trafficking. The 293F system was selected for its convenience under our experimental conditions, and we anticipated that the results would directly reflect LAT4 expression in the Xenopus oocyte system, given that Xenopus oocytes are also known to be a highly stable expression platform. However, we agree with the reviewer that direct measurement of LAT4 mutant surface expression in Xenopus oocytes provides a more accurate and rigorous approach for analyzing the defective activity of these mutations, and we have performed the measurements as described below.

The oocytes were disrupted by pipetting and then centrifuged at 8000 × g for 20 minutes to remove the cell nuclei and organelles. The supernatant was subsequently centrifuged at 100,000 × g for 1 hour to collect the cell membrane fraction. Cell membranes were solubilized in a buffer containing 150 mM NaCl, 20 mM HEPES (pH 7.4), 1% (w/v) LMNG, and 0.1% (w/v) CHS at 4 °C for 30 min. Protein concentrations were determined using a BCA kit according to the manufacturer's instructions (Thermo Fisher Scientific). Cell surface protein levels were normalized to the total protein concentration and further analyzed by western blotting.

2. CONCERN: HEK293F cells were transfected with HA-tagged wild-type and mutant hLAT4 constructs for surface labelling using PE-conjugated anti-HA tag antibody followed by flow cytometry to show proper surface expression of the studied mutants. There is no description of the location of the HA tag. Taking into account that both N- and C-terminal are intracellular, HA tag in the N- or C-term would be not detected by flow cytometry. Please, indicate the location of the HA tag in these studies. In general, it is a bizarre protocol because 3H-Met uptake was studied in Xenopus oocytes without tagged LAT4 and LAT4 surface expression in human cells with a tagged version.

Response: The HA tag was inserted between residues G62 and G63 of LAT4 in the extracellular loop and we have added this description in lines 443-444 as requested. We have additionally measured the surface expression of untagged LAT4 and LAT4 mutants in Xenopus oocytes to confirm that the observed activity defects are not due to compromised expression, as suggested by the reviewer.

3. Minor point: please, also indicate in the transport figures the concentration of 3H-Met used in these studies. The information is only found in Mat and Met section. Please, make the exercise of reading your paper easier to the readers.

Response: We appreciate the reviewer's suggestion and have added the concentration of [^{3}H]methionine to the legend of figures for clarity.

TBM-1 as a potential leading compound to develop a new cancer therapy.

1. CONCERN: The authors, as indicated above, show that TBM-1 inhibits traced 3H-Met uptake in oocytes expressing LAT4 with IC50 about 1000-fold lower that the general L-system transport inhibitor BCH. Also importantly, TBM-1 barely decreases the accumulation of 3H-Met in blood 1 h to 10 h after an oral gavage (from 500 CPM to 400 CPM from a basal of 50 CPM). What it is not shown is that administration of TBM-1 decreases Met concentration in plasma or blood? Surprisingly, the authors support the idea that the small effect of TBM-1 in 3H-Met absorption (gavage to blood) causes Met Restriction that results in the decrease of tumor progression (volume and weight) in MMTV-PyVT mice. I like the idea but the small effect on 3H-Met gavage accumulation in blood seems too small to decrease tumor progression and increase T cells in the tumor infiltrate showed in the manuscript. Plasma murine Met concentration is around 20 uM that can reach 5 uM with Met Restriction conditions. I ask the authors to show if TBM-1 treatment decrease Met concentration in plasma or blood of MMTV-PyVT mice comparable with the Met Restriction protocols affecting tumor growth quoted in the introduction.

Response: We agree with the reviewer that measuring the methionine (Met) concentration in plasma will better reflect the effect of TBM-1, and we have performed this measurement as requested. Eight-week-old female C57BL/6J mice were randomly assigned to three groups: control diet group, methionine-restriction diet group, and control diet TBM-1-treated group. Intraperitoneal injections were administered daily, with TBM-1-treated mice receiving TBM-1 at 0.5 mg/kg and control mice receiving vehicle (DMSO). Mice were fed either a control diet (containing 0.86% methionine without cystine) or a methionine-restricted diet (0.172% methionine without cystine) ad libitum for 8 days. Blood samples were collected from the retro-orbital sinus on days 0, 2, 5, 7 and 9. Plasma was obtained after centrifugation at 13,200 g for 10 minutes at 4 °C and subsequently analyzed for metabolomics profiling.

Mice fed a diet containing 0.86% methionine without cystine died within 9 days when treated with TBM-1 at doses exceeding 2 mg/kg/day; therefore, the TBM-1 dose was adjusted to 0.5 mg/kg/day. Plasma methionine levels measured after 5 h of fasting showed no significant differences between groups; accordingly, blood samples were collected without fasting. Data shown are means ± SD ($n \geq 5$ biological replicates). The results showed that plasma methionine levels were significantly reduced by the methionine-restricted (MR) diet and by TBM-1 treatment, confirming that TBM-1 effectively lowers plasma methionine levels. This effect was most pronounced on day 2, with differences diminishing at later time points, probably due to the frequent blood sample collection.

2. SUGGESTION: t-he abstract and the introduction highlight the overexpression of LAT4 in several cancer cells with the following scenario (line 81): "Cancer cells with increased LAT4 expression consumed and outcompeted T cells for methionine which in turn impaired T cell immunity by disrupting methionine metabolism, increasing PD-1 expression in tumor-infiltrating CD4+ 83 T cells and decreasing intracellular SAM in CD8+ 84 T cells (Bian et al., 2020, Pandit et al., 2023)". Does MMTV-PyVT tumor cells express LAT4? If these cells do express the transporter what is the direct impact of TBM-1 in these tumor cells? As clearly indicated in the literature quoted in the introduction LAT4 is a Met diffuser (i.e., net transport goes from a compartment of high concentration to another of lower concentration) that play a role in the basolateral efflux of Met (and Phe and Branched neutral amino acids- in agreement with the main proposition of the present manuscript. This transport mechanism differ from the other L system transporters (LAT1 and LAT2) that are exchangers (i.e., tertiary amino acid active transporters). In this scenario, considering that intracellular AA concentrations are larger than in plasma, one is forced to think that LAT4 in cancer cells would facilitate the efflux of Met and the other AA substrates from the tumor cells to the extracellular space. Then, what is the advantage for tumor cells to overexpress LAT4? Could the authors consider this issue in the discussion?

Response: We thank the reviewer for raising this important point. Determining whether LAT4 functions predominantly as an importer or exporter of methionine in vivo remains challenging, as it is difficult to selectively measure methionine levels in tumor tissue and distinguish them from methionine in surrounding non-tumor cells. Most published LAT4 methionine uptake inhibition studies were performed in vitro (Bian et al., 2020, PMID:32879489; Hong et al., 2025, PMID:39747898; Pandit et al., 2023, PMID:37147330). However, in previous studies, comparison of tumor volume and T-cell infiltration between SLC43A2 knockout and SLC43A2-intact tumors in tumor-bearing models support the hypothesis that LAT4 facilitates competition with T cells for methionine within the tumor microenvironment.

Previous studies on SLC43A2 (LAT4) have mainly used cancer cell lines with high LAT4 expression. In our study, MMTV-PyVT tumor cells expressed only detectable levels of LAT4, which were lower than LAT1. The MMTV-PyVT mouse model was chosen to evaluate whether TBM-1 could induce a systemic methionine reduction sufficient to suppress tumor progression in a solid tumor, such as breast cancer. This issue has been addressed in lines 337–342 of the revised Discussion.

3. SUGGESTION: Allosteric inhibitors have the advantage that do not compete with substrate to inhibit the transporter activity. Calculated TBM-1 binding site partially coincides substrate Phe binding site and also suggest that would block the cytosolic gate. Thus, TBM-1 could be a noncompetitive inhibitor of human LAT4. I suggest the authors to check whether TBM-1 would affect transport Vmax in a way largely independent of the substrate concentration.

Response: We thank the reviewer for this constructive suggestion. To assess whether TBM-1 affects the transport Vmax in a manner largely independent of substrate concentration, we measured the Vmax of methionine uptake by LAT4 in the presence or absence of 50 μM TBM-1. Oocytes were incubated in buffer containing Met (1 μCi) at the desired concentrations for 10 min at room temperature. Methionine uptake was calculated by subtracting background signal (oocyte without injection of LAT4 RNA) from WT (oocyte injected with LAT4 RNA) measurements. Data points are means ± SEM (n=3 biologically independent experiments). The results demonstrate that TBM-1 acts as a noncompetitive inhibitor of LAT4, with Km value and Vmax value of 8.39 mM and 2.23 nmol/min, for the TBM-1-free group and 8.21 and 0.63 nmol/min for the TBM-1-treated groups, respectively.

Referee #3:

The study by Ding et al. reports the first structures of the methionine transporter LAT4 (SLC43A2) in multiple states, and also the structure of ENBT1 (SLC43A3). Coupled with these structural studies, the authors use mutagenesis to tease apart the substrate binding and transport cycle of the proteins. Finally, the authors identify a saponin binding site in LAT4 and use this as the basis for a screen for inhibitors. They identify tubeimoside-1 (TBM-1) as a potential inhibitor, characterizing the activity of this molecule on methionine transport via cell and mouse studies. The structural and biochemical aspects of the study are well designed, executed, analyzed and presented. However, the characterization of TBM-1 as a LAT4 inhibitor is incomplete and therefore the interpretability of these results is uncertain. Furthermore, the structure of ENBT1 is included in this study, but the protein is not introduced or discussed in any detail. Therefore, the significance of this structure is unclear (and likely under-exploited), other than providing the outward-facing structure of a close paralog for analysis of the LAT4 structures.

This manuscript will require significant revision before publication, though ultimately I believe this is achievable. While the authors decision, I strongly encourage removing the mouse studies that are of questionable value, and adding an analysis of ENBT1.

We sincerely thank the reviewer for the thorough and insightful evaluation of our work, as well as for the thoughtful comments and valuable suggestions.

Critical

- The screen for saponins (Fig 5e) requires tests with and without LAT4 for each compound, as these molecules can non-specifically interact with the membrane and may each have unique off-targets.

Response: We thank the reviewer for this valuable suggestion and completely agree with the proposed approach. In fact, our previously presented results were calculated using exactly this method. In addition to their potential for non-specific inhibition, saponins can destabilize the oocyte membrane, which may interfere with accurate measurements of inhibitory effects, as observed in assays performed with 50 μM digitonin (Fig. B). For this reason, saponins were screened both with and without injection of LAT4 RNA. Residual methionine uptake was calculated as (WT + compound minus Control + compound) divided by (WT minus Control), as shown in Fig. a. Consistent with the reviewer's suggestion, any saponin that produced measurable effects in control oocytes was excluded from the analysis.

- The relatively low affinity of TBM-1 for LAT4, and the absence of a screen for off-target proteins (CETSA) and PK/PD data all make the mouse studies very difficult to interpret. It is unclear if LAT4 is the therapeutic target for the in vivo experiments, though this is implied. In fact, the authors note TBM-1 is already known to affect other pathways (Lines 327-329).

Response: We agree with the reviewer that TBM-1 may have additional molecular targets capable of inhibiting cancer progression. For this reason, we only propose TBM-1 as a prototype compound for further validation. In our previous experiments, the inhibitory effect of TBM-1 on LAT4 was confirmed in vitro (Fig. 5E-G), and scRNA-seq analysis indicated that TBM-1 could impair methionine-associated metabolic pathways (Fig. 6D, E). To address the reviewer's suggestion, we also measured plasma methionine levels after TBM-1 treatment and compared them with those in the MR-diet group. These results suggest that TBM-1 is likely to inhibit tumor progression by reducing plasma Met levels and thereby modulating methionine metabolism in different cell types within the tumor microenvironment, at least in part through targeting LAT4.

Major

- ENBT1 is only mentioned briefly in the abstract, and not noted at all in the introduction. The background of this protein should be discussed, along with its relevance to LAT4.

Response: We thank the reviewer for highlighting this omission. In the revised manuscript, we have added the background of ENBT1 and its relevance to LAT4 in lines 75–81 as requested. ENBT1 and LAT4 are both members of the SLC43 family. In this study, the experimentally resolved structure of ENBT1 in the outward-facing conformation was used solely to identify residues important for conformational transitions of LAT4, by comparison with inward-facing LAT4 structure. We chose ENBT1 for this purpose because its experimentally determined structure provides a more persuasive basis than a purely predicted outward-facing LAT4 model. The detailed structural features of ENBT1 will be described in our future work.

Minor

- (Line 114) How are nanodiscs meant to help avoid preferred orientation?

Response: Comparing LAT4 embedded in LMNG micelles with LAT4 reconstituted into nanodiscs, the nanodisc environment probably reduces particle adsorption to the air–water interface by shielding hydrophobic and highly charged regions from direct exposure. However, reconstitution into nanodiscs alone is not sufficient to fully resolve the issue; reduced-graphene–coated grids also need to be used.

- (Lines 172-173) It is odd to assert Phe and Met share a binding pocket when the residues shown in Fig. 3a and 3c are different. Perhaps show an overlay of the substrate bound-structures, combine Fig 3b and 3d, and/or make an equivalent of Fig 3e for the Phe binding.

Response: Alignment of Phe- and Met-bound structures obtained after a 200 ns molecular dynamics simulation indicates that Met and Phe bind at similar sites, as shown below. In response to the reviewer's suggestion, we have also provided a figure equivalent to Fig. 3C for the Phe binding.

- (Line 184) The authors assert these mutations alter the substrate permeation pathway. It would be helpful to clearly label these locations on the protein model.

Response: In response to the reviewer's suggestion, we have added Fig. 3hH and Fig. 3I, in which the residues comprising the binding pocket and those along the substrate permeation pathway are colored green and blue, respectively, consistent with the color scheme shown in Fig. 3G.

- (Line 201) I am not clear what states are being compared. Is this bound vs apo, or LAT1-IF-apo vs ENBT1-OF-apo?

Response: The comparison is between $LAT4_{APO}$ and $ENBT1_{APO}$. We have revised lines 202–203 to clarify this point.

- (Lines 229-231) The mention of pH sensitive MFS is unexpected and confusing. Has it been shown if LAT4 is (or is not) pH sensitive? This is only relevant if LAT4 has been demonstrated NOT to be pH sensitive, and if pH sensitive this could arise from residues outside this region.

Response: We have added a description of the pH-insensitive property of LAT4 and cited the relevant reference in line 231-234 to clarify this point as suggested.

- (Line 246) The authors assert that digitonin binds stably in the MD simulations. However, this does not appear supported by the data. Fig S5d shows an increasing RMSD, while the RMSD for substrates plateaus.

Response: We agree that the original presentation of Appendix Fig. S5D could be interpreted as showing an increasing RMSD. We have revised Appendix Fig. S5D by replacing it with data from an additional 200 ns MD simulation no longer exhibits a continuously increasing RMSD.

- (Line 388) The lipid mix should be specified.

Response: The lipid mix (POPC:POPE:POPG = 3:1:1) has been specified in line 397 as requested.

- How was the Met placed for MD?

Response: Met was positioned in the same binding site as Phe in the $LAT4_{PHE}$ structure for the MD simulations.

- I find Figs 1e, 1f, 2f, 4c, and 4d hard to interpret due to showing the protein surface as empty shell for slice and also the secondary structure. I suggest removing the secondary structure model and visualizing this volume as a slice through a solid object with neutral interior color such as grey.

Response: We have removed the secondary structure and presented the protein surface as a slice with a neutral grey interior in Figs. 1E, 1F, 4C, and 4D, as suggested. For Fig. 2F, using a grey interior would obscure the hydrophobic feature mapping and the visual distinction between the C-lobe and N-lobe.

- It is difficult to see the hydrophobicity of the LAT4 binding site in Fig 2f. I suggest zooming into this region.

Response: We have zoomed in on the view of the PHE binding site in Fig. 2F as requested.

- The fraction of particles that are use in the final reconstructions is relatively small. Are the excluded particles "junk" or genuine protein but conformationally heterogenous? And if flexible, did the authors attempt to model this flexibility using available methods?

Response: Most of the excluded particles are junk, exhibiting less well-defined structural features after classification and reconstruction.

Reference:

Bian, Y., Li, W., Kremer, D.M., Sajjakulnukit, P., Li, S., Crespo, J., Nwosu, Z.C., Zhang, L., Czerwonka, A., Pawlowska, A., et al. (2020). Cancer SLC43A2 alters T cell methionine metabolism and histone methylation. Nature 585, 277-282.

Guetg, A., Mariotta, L., Bock, L., Herzog, B., Fingerhut, R., Camargo, S.M., and Verrey, F. (2015). Essential amino acid transporter Lat4 (Slc43a2) is required for mouse development. J Physiol 593, 1273-1289.

Hong, X.L., Huang, C.K., Qian, H., Ding, C.H., Liu, F., Hong, H.Y., Liu, S.Q., Wu, S.H., Zhang, X., and Xie, W.F. (2025). Positive feedback between arginine methylation of YAP and methionine transporter SLC43A2 drives anticancer drug resistance. Nat Commun 16, 87.

Mastrototaro, L., Sponder, G., Saremi, B., and Aschenbach, J.R. (2016). Gastrointestinal methionine shuttle: Priority handling of precious goods. IUBMB Life 68, 924-934.

Pandit, M., Kil, Y.S., Ahn, J.H., Pokhrel, R.H., Gu, Y., Mishra, S., Han, Y., Ouh, Y.T., Kang, B., Jeong, M.S., et al. (2023). Methionine consumption by cancer cells drives a progressive upregulation of PD-1 expression in CD4 T cells. Nat Commun 14, 2593.

1st Revision - Editorial Decision **20th Feb 2026**

Dear Dr. Yin,

Thank you for submitting the revised version of your manuscript to The EMBO Journal The study has now been seen by two of the original referees, and I have included their comments below.

While reviewer #3 is satisfied with the revision, reviewer #2 finds that the provided evidence for the role of TBM-1 in suppression of tumour growth via regulation of decreased intestinal absorption of methionine. The reviewer proposes a potential alternative mechanism, in which TBM-1 might block efflux of methionine from T cells, thus increasing their anti-tumor immune response. Please address this remaining concern either via the proposed analysis of transcriptomic data, or by toning down of the conclusions and discussion of the alternative modes of the mechanism of TBM-1 action. Please also add this more detailed consideration and discussion of the mode of tBM-1 action in the manuscript text.

Additionally, there are several formatting and editorial aspects that would need to be addressed in the revised version:

1. Please note that it is The EMBO Journal policy for the transcript of the editorial process (containing referee reports and your response letters) to be published as an online supplement to each paper. If you should prefer removal of any referee-only figures included in the point-by-point response(s), e.g. because they may still be used for future publication or because they have been reproduced from published work by others, please do let us know immediately via response email.
More information is available here: https://www.embopress.org/transparent-process#Review_Process
2. Please ensure that the funding information is correct and identical both in the manuscript and our online system. Currently, Shenzhen Basic Research Key Project (JCYJ20220818102811024) is not mentioned in our online system.
3. CRediT has replaced the traditional author contributions section because it offers a systematic, machine-readable author contributions format that allows for more effective research assessment. Please remove the Authors Contributions from the manuscript and use the free text boxes beneath each contributing author's name in our online submission system to add specific details on the author's contribution. More information is available in our guide to authors.
4. Please rename "Competing interests" section into "Disclosure and competing interests statement".
5. Please update references according to The EMBO Journal style - where there are more than 10 authors on a paper, the first 10 should be listed, followed by 'et al.'
6. Individual figure panels for Fig. 3H-I are currently not mentioned in the manuscript text. Please add the corresponding callouts.
7. In the figure legend for Figure 3I, please correct the label - current labelled with J instead of I.
8. Please remove main figure legends from the individual figure files, while leaving them in the manuscript text file.
9. Please remove the Appendix figure legends from the manuscript text file.
10. In the Appendix, please remove the author list and affiliations from the cover page.
11. Please rename "Source data for primer" into Table EV1 and add a brief legend in the table file.
12. In the Data Availability section, please add resolvable links to the EMD-61324, EMD-61325, EMD-61326, EMD-62783, 9JBS, 9JBT, 9JBU, 9L38, PRJCA038601 datasets. More information about the format of this section can be found here: https://link.springer.com/partners/embo-press/editorial-policies#Data%20availability%20statement.
13. Please upload source data as one folder per figure. Please also submit the Source Data Checklist, which can be downloaded here: https://www.embopress.org/pb-assets/embo-site/EMBOpress_Source_Data_Checklist.xlsx
14. In our standard image check, our software noted similarities between the two-dimensional class averages presented in Appendix Fig S2B and Appendix Fig S3B. A brief explanation would be very helpful - I assume this is due to the same molecule in the apo and ligand-bound state being analysed.
15. Papers published in The EMBO Journal are accompanied online by a 'Synopsis' to enhance discoverability of the manuscript. It consists of A) a short (1-2 sentences) summary of the findings and their significance, B) 3-4 bullet points highlighting key results and C) a synopsis image that is 550x300-600 pixels large (width x height, jpeg or png format). You can either show a model or key data in the synopsis image. Please note that the image size is rather small and that text needs to be readable at the final size. Please send us this information together with the revised manuscript.

We generally allow three months as standard revision time. Should you foresee a problem in meeting this deadline, please let us know in advance to discuss an extension.

As a matter of policy, competing manuscripts published during this period will not negatively impact on our assessment of the conceptual advance presented by your study. However, please contact me as soon as possible upon publication of any related work to discuss the appropriate course of action.

When preparing your letter of response to the referees' comments, please bear in mind that this will form part of the Review Process File and will therefore be available online to the community. For more details on our Transparent Editorial Process, please visit our website: https://www.embopress.org/page/journal/14602075/authorguide#transparentprocess. Please also see the attached document with instructions for guidelines on preparation of the revised manuscript.

Please feel free to contact me if have any questions regarding this final revision. Thank you again for giving us the chance to

consider your manuscript for The EMBO Journal. I look forward to receiving the final version.

With best wishes,

Ieva

Ieva Gailite, PhD
Senior Scientific Editor
The EMBO Journal
Meyerhofstrasse 1
D-69117 Heidelberg
Tel: +4962218891309
i.gailite@embojournal.org

Instructions for preparing your revised manuscript:

Read our guidance for manuscript revisions and related editorial policies: https://link.springer.com/journal/44318/submission-guidelines#cms-Revised-submissions

We realize that it is difficult to revise to a specific deadline. In the interest of protecting the conceptual advance provided by the work, we recommend a revision within 3 months (21st May 2026). Please discuss the revision progress ahead of this time with the editor if you require more time to complete the revisions.

Referee #2:

The manuscript represents a seminal work on the high resolution structure of human LAT4 and structure/functional relationship analysis bringing the first transport mechanisms of LAT4 as one of the three members of the SLC43 family. This by itself merit publication in EMBO J. If the authors consider reporting only the structural and related functional information this could be consider a straightforward major changes.

About the identification of TBM-1 as the best LAT4 inhibitor known I have some doubts because the authors went directly to test the effect of inhibiting LAT4 in tumor growth in mouse. The tumor model used presents low expression of LAT4, and because administration o TBM-1 results in decreased intestinal absorption of radiolabelled L-methionine after an oral gavage, the authors support the idea that methionine restriction might be the cause of the decreased tumor growth. The problem I see is that the impact of TBM-1 in intestinal absorption of L-methionine and in L-methionine plasma levels seem to be very mild (see 1. CONCERN below). In the 2. SUGGESTION point below, I suggest an alternative explanation: TBM-1 might block efflux of L-methionine from T cells increasing anti-tumor immune response resulting in decreased tumor growth. Most probably, the authors have scRNA seq data to test this possibility by looking at PD-1 expression, methionine metabolism and methylation processes. If the authors agree with this possibility, I consider these major changes.

Detailed review report about my previous points of the first revision

Structure and structure/functional relationship of human LAT4.
1 Concern. I am satisfied with the WB of LAT4 in purified membranes of Xenopus oocytes expressing WT or mutated versions of LAT4. I guess the protocol of oocyte membranes purification harvested total oocyte membranes, and not only cellular plasma membrane, but together with the surface expression in HEK293F cells it is clear that the transport defect of the studied mutations is not the consequence of defective cell surface expression. All these functional results validate the CryoEM structure of LAT4 bound to L-Phe and the structure/function relationship analysis.
2 Concern. Perfect. Location of the HA tag within Gly 62 and Gly 63 (clearly external) is now indicated in the manuscript.
3 Minor point. Concentration of 3H-Met used in the transport experiments is now indicated in the corresponding figures.
TBM-1 as a potential leading compound to develop a new cancer therapy.
1. CONCERN. In the previous version, the manuscript contains the very mild effect of TBM-1 administration in vivo to the radioactivity reaching plasma after a gavage administration of 3H-Met. I ask for the impact of TBM-1 on the concentration of methionine in plasma. The authors studied the impact on L-methionine concentration in plasma for 9 days of daily i.p. administration of one TBM-1 dose of 0.5 mg/kg BW. Interestingly, 2 days after treatment, plasma methionine concentration drops from 80% to 40% of the control diet values, and then there is a progressive recovery of methionine concentration that reached pre-treatment values at day 9. Interestingly, the same profile is obtained if mice are under methionine-restricted diet. The

authors argue that the drop at day 2 treatment values confirm the impact of the LAT4 inhibitor (TBM-1) on the intestinal absorption of methionine, and the ulterior recovery is the consequence of the additional blood sampling from retro-orbital sinus at days 5, 7 and 9. I do not follow how these blood sampling would recover plasma methionine concentration. How large was the sampled volume each day?
I have an alternative possible explanation. In the basolateral membrane of the mouse small intestine are also expressed LAT2/CD98hc and TAT1 that also contribute to the methionine absorption in the intestine (Rossier et al. J Biol Chem. 1999 Dec 3;274(49):34948-54. doi:10.1074/jbc.274.49.34948 ; Ramadan et al. J Cell Physiol. 2006 Mar;206(3):771-9. doi: 10.1002/jcp.20531). Does LAT4 inhibition and methionine-restricted diet upregulate the expression of LAT2? In this line, what is the protocol of the very mild decreased effect of TBM-1 on the radioactivity reaching plasma after a gavage of 3H-Met? Mice were treated for several days before the gavage was performed that would suggest again an adaptation to the defective methionine absorption? In this case, both Met restriction and TBM-1 effect results in a very mild limited systemic availability of L-methionine.
2. SUGGESTION. I agree, that the sense of transport with a uniporter like LAT4 depends on the relative concentration at both sides of the cell membrane, at present determination of the methionine concentration in the extracellular space, in the cytosol of tumor cells and T-cells seems not affordable. Then, the question is, how TBM-1 affects the competition for methionine between tumour cells and T cells described earlier (Bian et al., 2020; Bandit et al. 2023)? The authors indicate that MMTV-PyVT tumor cells expressed only detectable levels of LAT4, which were lower than LAT1. In contrast, mouse CD4T and CD8T cells express similarly LAT4 and LAT1 mRNAs. In this scenario, it could be expected a larger impact of TBM-1 in T cells than in the tumor cells. Thus, methionine might be uptake in exchange with other cellular amino acids by LAT1 and released to the extracellular space via LAT4. If this were the case, TMB-1 would increase methionine concentration in T cells that will result in better antitumor immunity causing decreased tumor growth, what it is observed (Fig. 6 A-C). If this possibility were correct transcriptomic data would show increased methionine metabolism and SAM methylation-related processes. The authors might have these data from the scRNA of T cells to refute this possibility.

3. SUGGESTION. The authors performed the kinetics of L-methionine uptake in oocytes showing the Vmax effect of TBM-1 as an allosteric inhibitor. This results strongly points to a TMB-1 binding site that will not compromise L-methionine binding, apparently matching the molecular docking of TBM-1 shown in Fig. S5 F. I suggest adding this result as a supplementary figure.

Referee #3:

The study by Ding et al. reports the first structures of the methionine transporter LAT4 (SLC43A2) in multiple states, and also the structure of ENBT1 (SLC43A3). Coupled with these structural studies, the authors use mutagenesis to tease apart the substrate binding and transport cycle of the proteins. Finally, the authors identify a saponin binding site in LAT4 and use this as the basis for a screen for inhibitors. They identify tubeimoside-1 (TBM-1) as a potential inhibitor, characterizing the activity of this molecule on methionine transport via cell and mouse studies. The structural and biochemical aspects of the study are well designed, executed, analyzed and presented.

The authors have done a commendable job addressing my previous concerns. I have identified one small error that should be corrected.

Minor
-There appears to be a typo in Fig. 3c, labeling Q335 as G335.

Point-by-point response to reviewers

We are grateful to the reviewers for their thorough evaluation of our manuscript and for their constructive suggestions. A detailed point-by-point response to all comments and suggestions of the reviewers are provided in this document.

Referee #2:

The manuscript represents a seminal work on the high resolution structure of human LAT4 and structure/functional relationship analysis bringing the first transport mechanisms of LAT4 as one of the three members of the SLC43 family. This by itself merit publication in EMBO J. If the authors consider reporting only the structural and related functional information this could be consider a straightforward major changes.

About the identification of TBM-1 as the best LAT4 inhibitor known I have some doubts because the authors went directly to test the effect of inhibiting LAT4 in tumor growth in mouse. The tumor model used presents low expression of LAT4, and because administration o TBM-1 results in decreased intestinal absorption of radiolabelled L-methionine after an oral gavage, the authors support the idea that methionine restriction might be the cause of the decreased tumor growth. The problem I see is that the impact of TBM-1 in intestinal absorption of L-methionine and in L-methionine plasma levels seem to be very mild (see 1. CONCERN below). In the 2. SUGGESTION point below, I suggest an alternative explanation: TBM-1 might block efflux of L-methionine from T cells increasing anti-tumor immune response resulting in decreased tumor growth. Most probably, the authors have scRNA seq data to test this possibility by looking at PD-1 expression, methionine metabolism and methylation processes. If the authors agree with this possibility, I consider these major changes.

We sincerely thank the reviewer for the careful and insightful evaluation of our manuscript. We greatly appreciate the thoughtful suggestions provided and we are grateful for the reviewer's continued engagement throughout the revision process.

Detailed review report about my previous points of the first revision

Structure and structure/functional relationship of human LAT4.

1 Concern. I am satisfied with the WB of LAT4 in purified membranes of Xenopus oocytes expressing WT or mutated versions of LAT4. I guess the protocol of oocyte membranes purification harvested total oocyte membranes, and not only cellular plasma membrane, but together with the surface expression in HEK293F cells it is clear that the transport defect of the studied mutations is not the consequence of defective cell surface expression. All these functional results validate the CryoEM structure of LAT4 bound to L-Phe and the structure/function relationship analysis.

2 Concern. Perfect. Location of the HA tag within Gly 62 and Gly 63 (clearly external) is now indicated in the manuscript.

3 Minor point. Concentration of 3H-Met used in the transport experiments is now indicated in the corresponding figures.

TBM-1 as a potential leading compound to develop a new cancer therapy.

1. CONCERN. In the previous version, the manuscript contains the very mild effect of TBM-1 administration in vivo to the radioactivity reaching plasma after a gavage administration of 3H-Met. I ask for the impact of TBM-1 on the concentration of methionine in plasma. The authors studied the impact on L-methionine concentration in plasma for 9 days of daily i.p. administration of one TBM-1 dose of 0.5 mg/kg BW. Interestingly, 2 days after treatment, plasma methionine concentration drops from 80% to 40% of the control diet values, and then there is a progressive recovery of methionine concentration that reached pre-treatment values at day 9. Interestingly, the same profile is obtained if mice are under methionine-restricted diet. The authors argue that the drop at day 2 treatment values confirm the impact of the LAT4 inhibitor (TBM-1) on the intestinal absorption of methionine, and the ulterior recovery is the consequence of the additional blood sampling from retro-orbital sinus at days 5, 7 and 9. I do not follow how these blood sampling would recover plasma methionine concentration. How large was the sampled volume each day?

I have an alternative possible explanation. In the basolateral membrane of the mouse small intestine are also expressed LAT2/CD98hc and TAT1 that also contribute to the methionine absorption in the intestine (Rossier et al. J Biol Chem. 1999 Dec 3;274(49):34948-54. doi:10.1074/jbc.274.49.34948 ; Ramadan et al. J Cell Physiol. 2006 Mar;206(3):771-9. doi: 10.1002/jcp.20531). Does LAT4 inhibition and methionine-restricted diet upregulate the expression of LAT2? In this line, what is the protocol of the very mild decreased effect of TBM-1 on the radioactivity reaching plasma after a gavage of 3H-Met? Mice were treated for several days before the gavage was performed that would suggest again an adaptation to the defective methionine absorption? In this case, both Met restriction and TBM-1 effect results in a very mild limited systemic availability of L-methionine.

Response: We thank the reviewer for this thoughtful and detailed comment. The volume of blood collected at each time point was approximately 300 μl per mouse. We agree that repeated blood sampling itself is unlikely to account for the apparent recovery of plasma methionine levels observed at later time points. Instead, the attenuation of the observed effect may reflect nutritional stress in the control group rather than a true recovery of plasma methionine levels in the MR- and TBM-1-treated groups.

Regarding potential compensatory mechanisms, we acknowledge that LAT2/CD98hc and TAT1 are also expressed at the basolateral membrane of the small intestine and may contribute to intestinal methionine transport. However, this was not directly assessed in the current study.

With respect to the oral gavage experiment using [^{3}H]methionine, mice were not pre-treated with TBM-1 prior to the gavage. We agree with the reviewer that the very mild decrease in plasma radioactivity does not provide strong evidence for a robust inhibition of intestinal methionine absorption by TBM-1 in vivo, and the underlying reason for this modest effect remains unclear. In light of these considerations, and to avoid overinterpretation, we have removed this dataset from the revised manuscript.

2. SUGGESTION. I agree, that the sense of transport with a uniporter like LAT4 depends on the relative concentration at both sides of the cell membrane, at present determination of the

methionine concentration in the extracellular space, in the cytosol of tumor cells and T-cells seems not affordable. Then, the question is, how TBM-1 affects the competition for methionine between tumour cells and T cells described earlier (Bian et al., 2020; Bandit et al. 2023)? The authors indicate that MMTV-PyVT tumor cells expressed only detectable levels of LAT4, which were lower than LAT1. In contrast, mouse CD4T and CD8T cells express similarly LAT4 and LAT1 mRNAs. In this scenario, it could be expected a larger impact of TBM-1 in T cells than in the tumor cells. Thus, methionine might be uptake in exchange with other cellular amino acids by LAT1 and released to the extracellular space via LAT4. If this were the case, TMB-1 would increase methionine concentration in T cells that will result in better antitumor immunity causing decreased tumor growth, what it is observed (Fig. 6 A-C). If this possibility were correct transcriptomic data would show increased methionine metabolism and SAM methylation-related processes. The authors might have these data from the scRNA of T cells to refute this possibility.

Response: We thank the reviewer for this insightful suggestion. To test whether TBM-1 might enhance T cell anti-tumor immunity via methionine metabolism, we analyzed our scRNA-seq data focusing on T cells.

First, we examined canonical checkpoint genes (Pdcd1, Ctla4, Tigit, Lag3) and found they were consistently increased in T cells from TBM-1-treated tumors compared to controls. As checkpoint molecules can be induced upon activation, we further assessed effector genes (Cd69, Cd28, Prf1, Il2ra) and observed that these were also significantly upregulated in the TBM-1 group.

Next, we specifically analyzed methionine metabolism and methylation processes in $CD8^+$ T cells. Gene set enrichment analysis revealed that pathways including “methionine biosynthetic process”, “methionine metabolic process”, and “one-carbon metabolic process” were significantly enriched in $CD8^+$ T cells following TBM-1 treatment.

Collectively, these findings—the coordinated upregulation of both checkpoint and effector genes, along with the enrichment of methionine metabolism pathways—are supportive of the reviewer's hypothesis that TBM-1 may modulate T cell function through this mechanism. However, we also noted that the proportion of T cells in the MMTV-PyVT breast cancer tissues is relatively low compared to innate immune cells in our model. Therefore, while T cells exhibit signs of activation and metabolic reprogramming, their limited abundance led us to propose that myeloid cells may play a more dominant role in the observed anti-tumor phenotype.

3. SUGGESTION. The authors performed the kinetics of L-methionine uptake in oocytes showing the Vmax effect of TBM-1 as an allosteric inhibitor. This results strongly points to a TMB-1 binding site that will not compromise L-methionine binding, apparently matching the molecular docking of TBM-1 shown in Fig. S5 F. I suggest adding this result as a supplementary figure.

Response: We agree with the reviewer’s suggestion and have added the methionine uptake kinetics in oocytes as Appendix Figure. S5G.

Referee #3:

The study by Ding et al. reports the first structures of the methionine transporter LAT4 (SLC43A2) in multiple states, and also the structure of ENBT1 (SLC43A3). Coupled with these structural studies, the authors use mutagenesis to tease apart the substrate binding and transport cycle of the proteins. Finally, the authors identify a saponin binding site in LAT4 and use this as the basis for a screen for inhibitors. They identify tubeimoside-1 (TBM-1) as a potential inhibitor, characterizing the activity of this molecule on methionine transport via cell and mouse studies. The structural and biochemical aspects of the study are well designed, executed, analyzed and presented.

The authors have done a commendable job addressing my previous concerns. I have identified one small error that should be corrected.

We greatly appreciate the reviewer's thorough evaluation of our manuscript and the positive assessment of our study, and we thank the reviewer for the continued support throughout the review process.

Minor

-There appears to be a typo in Fig. 3c, labeling Q335 as G335.

Response: We appreciate the reviewer for pointing out this typographical error, and we have corrected the labeling to Q335 in Fig. 3c.

2nd Revision - Editorial Decision **27th Mar 2026**

Dear Dr. Yin,

Thank you for incorporating the final editorial and formatting requests in the manuscript. I am now pleased to inform you that your manuscript has been accepted for publication. Congratulations with a nice study!

Before we forward your manuscript to our publishers, we would like to propose some edits in the manuscript abstract and synopsis. I have also written a short blurb that will accompany the title of your manuscript in our online table of contents. Please take a look at the proposed text changes in the attached text file and let me know if any corrections are needed.

Your manuscript will be processed for publication by EMBO Press. It will be copy edited and you will receive page proofs prior to publication. Please note that you will be contacted by Springer Nature Author Services to complete licensing and payment information.

You may qualify for financial assistance for your publication charges - either via a Springer Nature fully open access agreement or an EMBO initiative. Check your eligibility: https://link.springer.com/journal/44318/how-to-publish-with-us

Should you be planning a Press Release on your article, please get in contact with embo_production@springernature.com as early as possible in order to coordinate publication and release dates.

If you have any questions, please do not hesitate to contact the Editorial Office. Thank you for this interesting contribution to The EMBO Journal!

Best wishes,

Ieva

Ieva Gailite, PhD
Senior Scientific Editor
The EMBO Journal
Meyerhofstrasse 1
D-69117 Heidelberg
Tel: +4962218891309
i.gailite@embojournal.org

*****
Please note that it is The EMBO Journal policy for the transcript of the editorial process (containing referee reports and your response letters) to be published as an online supplement to each paper. If you should prefer removal of any referee-only figures included in the point-by-point response(s), e.g. because they may still be used for future publication or because they have been reproduced from published work by others, please do let us know immediately via response email.
More information is available here: https://link.springer.com/partners/embo-press/editorial-policies#Peer%20review

EMBO Press Author Checklist

Corresponding Author Name: Yuxin Yin	
Journal Submitted to: The EMBO Journal	
Manuscript Number: EMBOJ-2025-122196	

USEFUL LINKS FOR COMPLETING THIS FORM
The EMBO Journal - Author Guidelines
EMBO Reports - Author Guidelines
Molecular Systems Biology - Author Guidelines
EMBO Molecular Medicine - Author Guidelines

Reporting Checklist for Life Science Articles (updated January
This checklist is adapted from Materials Design Analysis Reporting (MDAR) Checklist for Authors. MDAR establishes a minimum set of requirements in transparent reporting in the life sciences (see Statement of Task: 10.31222/osf.io/9sm4x). Please follow the journal's guidelines in preparing your
Please note that a copy of this checklist will be published alongside your article.

Abridged guidelines for figures
1. Data
The data shown in figures should satisfy the following conditions:
- the data were obtained and processed according to the field's best practice and are presented to reflect the results of the experiments in an accurate and unbiased manner.
- ideally, figure panels should include only measurements that are directly comparable to each other and obtained with the same assay.
- plots include clearly labeled error bars for independent experiments and sample sizes. Unless justified, error bars should not be shown for technical
- if n<5, the individual data points from each experiment should be plotted. Any statistical test employed should be justified.
- Source Data should be included to report the data underlying figures according to the guidelines set out in the authorship guidelines on Data

2. Captions
Each figure caption should contain the following information, for each panel where they are relevant:
- a specification of the experimental system investigated (eg cell line, species name).
- the assay(s) and method(s) used to carry out the reported observations and measurements.
- an explicit mention of the biological and chemical entity(ies) that are being measured.
- an explicit mention of the biological and chemical entity(ies) that are altered/varied/perturbed in a controlled manner.
- the exact sample size (n) for each experimental group/condition, given as a number, not a range;
- a description of the sample collection allowing the reader to understand whether the samples represent technical or biological replicates (including how many animals, litters, cultures, etc.).
- a statement of how many times the experiment shown was independently replicated in the laboratory.
- definitions of statistical methods and measures:
  - common tests, such as t-test (please specify whether paired vs. unpaired), simple χ2 tests, Wilcoxon and Mann-Whitney tests, can be unambiguously identified by name only, but more complex techniques should be described in the methods section;
  - are tests one-sided or two-sided?
  - are there adjustments for multiple comparisons?
  - exact statistical test results, e.g., P values = x but not P values < x;
  - definition of 'center values' as median or average;
  - definition of error bars as s.d. or s.e.m.

Please complete ALL of the questions below.
Select "Not Applicable" only when the requested information is not relevant for your study.

Materials

Newly Created Materials	Information included in the manuscript?	In which section is the information available? (Reagents and Tools Table, Materials and Methods, Figures, Data Availability Section)
New materials and reagents need to be available; do any restrictions apply?	Yes	Reagents and Tools Table

Antibodies	Information included in the manuscript?	In which section is the information available? (Reagents and Tools Table, Materials and Methods, Figures, Data Availability Section)
For **antibodies** provide the following information:<br>- Commercial antibodies: RRID (if possible) or supplier name, catalogue number and or/clone number<br>- Non-commercial: RRID or citation	Yes	Reagents and Tools Table

DNA and RNA sequences	Information included in the manuscript?	In which section is the information available? (Reagents and Tools Table, Materials and Methods, Figures, Data Availability Section)
**Short novel DNA or RNA including primers, probes**: provide the sequences.	Yes	Source Data Table

Cell materials	Information included in the manuscript?	In which section is the information available? (Reagents and Tools Table, Materials and Methods, Figures, Data Availability Section)
**Cell lines:** Provide species information, strain. Provide accession number in repository **OR** supplier name, catalog number, clone number, and/**OR** RRID.	Yes	Reagents and Tools Table
**Primary cultures:** Provide species, strain, sex of origin, genetic modification status.	Yes	Materials and Methods
Report if the cell lines were recently **authenticated** (e.g., by STR profiling) and tested for mycoplasma contamination.	Yes	Materials and Methods

Experimental animals	Information included in the manuscript?	In which section is the information available? (Reagents and Tools Table, Materials and Methods, Figures, Data Availability Section)
**Laboratory animals or Model organisms:** Provide species, strain, sex, age, genetic modification status. Provide accession number in repository **OR** supplier name, catalog number, clone number, **OR** RRID.	Yes	Materials and Methods
**Animal observed in or captured from the field:** Provide species, sex, and age where possible.	Not Applicable	
Please detail **housing and husbandry conditions**.	Yes	Materials and Methods

Plants and microbes	Information included in the manuscript?	In which section is the information available? (Reagents and Tools Table, Materials and Methods, Figures, Data Availability Section)
**Plants:** provide species and strain, ecotype and cultivar where relevant, unique accession number if available, and source (including location for collected wild specimens).	Not Applicable	
**Microbes:** provide species and strain, unique accession number if available, and source.	Yes	Materials and Methods

Human research participants	Information included in the manuscript?	In which section is the information available? (Reagents and Tools Table, Materials and Methods, Figures, Data Availability Section)
If collected and within the bounds of privacy constraints report on age, sex and gender or ethnicity for all study participants.	Not Applicable	

Core facilities	Information included in the manuscript?	In which section is the information available? (Reagents and Tools Table, Materials and Methods, Figures, Data Availability Section)
If your work benefited from core facilities, was their service mentioned in the acknowledgments section?	Yes	Acknowledgments

Design

Study protocol	Information included in the manuscript?	In which section is the information available? (Reagents and Tools Table, Materials and Methods, Figures, Data Availability Section)
If study protocol has been **pre-registered, provide DOI in the manuscript**. For clinical trials, provide the trial registration number **OR** cite DOI.	Not Applicable	
Report the **clinical trial registration number** (at ClinicalTrials.gov or equivalent), where applicable.	Not Applicable	

Laboratory protocol	Information included in the manuscript?	In which section is the information available? (Reagents and Tools Table, Materials and Methods, Figures, Data Availability Section)
Provide DOI OR other citation details if **external detailed step-by-step protocols** are available.	Not Applicable	

Experimental study design and statistics	Information included in the manuscript?	In which section is the information available? (Reagents and Tools Table, Materials and Methods, Figures, Data Availability Section)
Include a statement about **sample size** estimate even if no statistical methods were used.	Yes	Materials and Methods
Were any steps taken to minimize the effects of subjective bias when allocating animals/samples to treatment (e.g. **randomization procedure**)? If yes, have they been described?	Yes	Materials and Methods
Include a statement about **blinding** even if no blinding was done.	Not Applicable	
Describe **inclusion/exclusion criteria** if samples or animals were excluded from the analysis. Were the criteria pre-established? If sample or data points were omitted from analysis, report if this was due to attrition or intentional exclusion and provide justification.	Yes	Materials and Methods
For every figure, are **statistical tests** justified as appropriate? Do the data meet the assumptions of the tests (e.g., normal distribution)? Describe any methods used to assess it. Is there an estimate of variation within each group of data? Is the variance similar between the groups that are being statistically compared?	Yes	Materials and Methods

Sample definition and in-laboratory replication	Information included in the manuscript?	In which section is the information available? (Reagents and Tools Table, Materials and Methods, Figures, Data Availability Section)
In the figure legends: state number of times the experiment was **replicated** in laboratory.	Yes	Figures
In the figure legends: define whether data describe **technical or biological replicates**.	Yes	Figures

Ethics

Ethics	Information included in the manuscript?	In which section is the information available? (Reagents and Tools Table, Materials and Methods, Figures, Data Availability Section)
Studies involving **human participants**: State details of **authority granting ethics approval** (IRB or equivalent committee(s), provide reference number for approval.	Not Applicable	
Studies involving **human participants**: Include a statement confirming that **informed consent** was obtained from all subjects and that the experiments conformed to the principles set out in the WMA Declaration of Helsinki and the Department of Health and Human Services Belmont Report.	Not Applicable	
Studies involving **human participants: For publication of patient photos**, include a statement confirming that consent to publish was obtained.	Not Applicable	
Studies involving experimental **animals**: State details of **authority granting ethics approval** (IRB or equivalent committee(s), provide reference number for approval. Include a statement of compliance with ethical regulations.	Yes	Materials and Methods
Studies involving **specimen and field samples:** State if relevant **permits** obtained, provide details of authority approving study; if none were required, explain why.	Not Applicable	

Dual Use Research of Concern (DURC)	Information included in the manuscript?	In which section is the information available? (Reagents and Tools Table, Materials and Methods, Figures, Data Availability Section)
Could your study fall under dual use research restrictions? Please check biosecurity documents and list of **select agents and toxins** (CDC): https://www.selectagents.gov/sat/list.htm	Not Applicable	
If you used a select agent, is the security level of the lab appropriate and reported in the manuscript?	Not Applicable	
If a study is subject to dual use research of concern regulations, is the name of the **authority granting approval and reference number** for the regulatory approval provided in the manuscript?	Not Applicable	

Reporting

The MDAR framework recommends adoption of discipline-specific guidelines, established and endorsed through community initiatives. Journals have their own policy about requiring specific guidelines and recommendations to complement MDAR.

Adherence to community standards	Information included in the manuscript?	In which section is the information available? (Reagents and Tools Table, Materials and Methods, Figures, Data Availability Section)
State if relevant guidelines or checklists (e.g., **ICMJE, MIBBI, ARRIVE, PRISMA**) have been followed or provided.	Not Applicable	
For **tumor marker prognostic studies,** we recommend that you follow the **REMARK** reporting guidelines (see link list at top right). See author guidelines, under 'Reporting Guidelines'. Please confirm you have followed these guidelines.	Not Applicable	
For **phase II and III randomized controlled trials**, please refer to the **CONSORT** flow diagram (see link list at top right) and submit the CONSORT checklist (see link list at top right) with your submission. See author guidelines, under 'Reporting Guidelines'. Please confirm you have submitted this list.	Not Applicable	

Data Availability

Data availability	Information included in the manuscript?	In which section is the information available? (Reagents and Tools Table, Materials and Methods, Figures, Data Availability Section)
Have **primary datasets** been deposited according to the journal's guidelines (see 'Data Deposition' section) and the respective accession numbers provided in the Data Availability Section?	Yes	Data Availability Section
Were **human clinical and genomic datasets** deposited in a public access-controlled repository in accordance to ethical obligations to the patients and to the applicable consent agreement?	Not Applicable	
Are **computational models** that are central and integral to a study available without restrictions in a machine-readable form? Were the relevant accession numbers or links provided?	Not Applicable	
If publicly available data were reused, provide the respective **data citations in the reference list**.	Not Applicable	
